# Category-selective deficits are the exception and not the rule: Evidence from a case-series of 64 patients with ventral occipito-temporal cortex damage

**DOI:** 10.1016/j.cortex.2021.01.021

**Published:** 2021-05

**Authors:** Grace E. Rice, Sheila J. Kerry, Ro J. Robotham, Alex P. Leff, Matthew A. Lambon Ralph, Randi Starrfelt

**Affiliations:** a MRC Cognition and Brain Sciences Unit (CBU), University of Cambridge, UK; b University College London Queen Square Institute of Neurology, UK; c Department of Psychology, University of Copenhagen, Denmark

**Keywords:** Face recognition, Word recognition, Stroke, Visual perception, Pure alexia, Prosopagnosia, Visual agnosia

## Abstract

The organisational principles of the visual ventral stream are still highly debated, particularly the relative association/dissociation between word and face recognition and the degree of lateralisation of the underlying processes. Reports of dissociations between word and face recognition stem from single case-studies of category selective impairments, and neuroimaging investigations of healthy participants. Despite the historical reliance on single case-studies, more recent group studies have highlighted a greater commonality between word and face recognition. Studying individual patients with rare selective deficits misses (a) important variability between patients, (b) systematic associations between task performance, and (c) patients with mild, severe and/or non-selective impairments; meaning that the full spectrum of deficits is unknown. The Back of the Brain project assessed the range and specificity of visual perceptual impairment in 64 patients with posterior cerebral artery stroke recruited based on lesion localization and not behavioural performance. Word, object, and face processing were measured with comparable tests across different levels of processing to investigate associations and dissociations across domains. We present two complementary analyses of the extensive behavioural battery: (1) a data-driven analysis of the whole patient group, and (2) a single-subject case-series analysis testing for deficits and dissociations in each individual patient. In both analyses, the general organisational principle was of associations between words, objects, and faces even following unilateral lesions. The majority of patients either showed deficits across all domains or in no domain, suggesting a spectrum of visuo-perceptual deficits post stroke. Dissociations were observed, but they were the exception and not the rule: Category-selective impairments were found in only a minority of patients, all of whom showed disproportionate deficits for words. Interestingly, such selective word impairments were found following both left and right hemisphere lesions. This large-scale investigation of posterior cerebral artery stroke patients highlights the bilateral representation of visual perceptual function.

## 1 Introduction

Strong hypotheses about the cerebral localisation, lateralisation, and selectivity of word and face processing have been proposed based on neuropsychological single case-studies and functional imaging in healthy subjects (Cohen et al., 2002; Ellis & Florence, 1990; Farah, 1991; Kanwisher et al., 1997; Rossion et al., 2003). Patients with pure alexia present with deficits in reading in the absence of deficits in writing or language (Gaillard et al., 2006; Starrfelt & Shallice, 2014). This deficit is specific to written words, and patients may have no deficits in object or face recognition. Pure alexia normally follows unilateral left ventral occipito-temporal (vOTC) damage (Leff et al., 2006; Starrfelt et al., 2009). In contrast, patients with (pure) prosopagnosia have impaired face recognition, in the absence of low-level visual processing impairments, and normal word and object recognition (Rossion, 2018; Susilo et al., 2015). Prosopagnosia typically follows right hemisphere or bilateral vOTC damage (Barton et al., 2002; Van Belle et al., 2011). Early reviews of such single case studies showed relative independence between word and face recognition (Farah, 1991, 1992, 2004, p. 1992), with object recognition falling in between, which is still reflected in current textbook knowledge (Gazzaniga et al., 2019). However, research on alexia/reading and prosopagnosia/face recognition have traditionally been rather separate fields, and direct comparisons of face and word recognition in the same patient are rare.

In contrast to the single-case literature, recent studies with larger samples have highlighted a greater degree of commonality/association between word and face recognition following unilateral stroke (Asperud et al., 2019; Gerlach et al., 2014; Martinaud et al., 2012). In one influential case-series investigation (in which multiple patients are studied in the same level of detail as a single-case study), Behrmann and Plaut (2014) reported that patients with seemingly pure deficits in face recognition or word recognition also showed measurable impairments in the other domain (albeit it to a lesser degree), when sensitive testing was used. The authors concluded that word and face recognition were underpinned by a primarily bilateral system, and thus unilateral damage to either hemisphere in the vOTC should result in a measurable impairment in both domains (Behrmann & Plaut, 2013, 2014). This finding of (mild) face recognition deficits in pure alexia was also found in a larger independent case-series analysis of patients with focal left hemisphere lesions to the posterior ventral temporal lobes (Roberts et al., 2013). Additional evidence for association between word and face recognition comes from studies showing impaired face processing abilities in developmental dyslexia (Collins et al., 2017; Gabay et al., 2017; Sigurdardottir et al., 2015). With this recent focus on distributed, bilateral networks in visual object recognition (Behrmann & Plaut, 2013, 2020), the selectivity and lateralization of word and face recognition is again hotly debated (see e.g., Geskin & Behrmann, 2018 and accompanying commentaries), and direct comparisons of patient performance in the two domains more frequent (see Behrmann & Plaut, 2020; Robotham & Starrfelt, 2017b for reviews).

Currently there is a trade-off between single case-studies using sensitive and in-depth testing, and group studies with more representative samples of patients but often with less sensitive testing. This trade-off gives rise to three critical issues that need to be resolved in order to assess whether visual perceptual deficits may be specific to one visual category or not, and whether there is a systematic relationship between performance in different categories. The first issue is patient recruitment. Patients are often recruited based on behaviour (e.g., a deficit in word recognition), and then that behaviour is assessed in-depth to determine at what level the impairment lies. However, patients’ performance in other domains (e.g., face recognition) are rarely tested in the same level of detail. This reliance on recruiting based on behaviour also means that it is unknown whether a similar lesion could give rise to no behavioural impairment (Leff & Starrfelt, 2014). Related to the point above, the second issue is that there may be a bias in the literature relating to patient severity. By definition, recruiting patients based on behaviour excludes any patient with no reportable impairment (which is an issue because the inclusion of mild patients provides important information regarding the necessity of a brain region in a given function). Similarly, recruiting based on (category-selective) behaviour also excludes patients who have non-selective deficits. These sources of recruitment biases in the single-case literature mean that the full spectrum of visual perceptual deficits following posterior cerebral artery stroke has yet to be fully elucidated in the same sample. A key contribution from a broader sample is the possibility of detecting systematic associations between functions. While association or co-occurrence of deficits in any given patient might be explained by collateral damage to neighbouring regions, systematic association of functions across patients might point to common underlying mechanisms. The final issue in the literature is a lack of control group data to gauge the range of individual variability in the domain of interest, and, even more importantly, the relationship in performance across domains which is the basis for determining the presence of a dissociation (Crawford et al., 2011; Gerlach et al., 2018). Therefore, despite the wealth of literature on face and word recognition after brain injury, comparing dissociations/associations across different studies (each with different methodologies/test batteries, patients and controls) is practically impossible (Robotham & Starrfelt, 2017b).

Here, we present the Back of the Brain project (BoB), which represents the largest and most in-depth database of higher-level vision following posterior cerebral artery stroke. The onus for the BoB project was on preserving the breadth of patients and the depth of behavioural testing, in order to assess both the range and specificity of visual perceptual functions post-stroke. A large-scale case-series approach was used to preserve the depth and sensitivity of testing found in single case-studies but also retain the breadth of patients obtained from group studies. We recruited 64 cases of posterior cerebral artery stroke (32 left hemisphere, 23 right hemisphere, 9 bilateral) based on lesion location, and not behavioural profile (for similar approaches see: Asperud et al., 2019; Butler et al., 2014; Gerlach et al., 2014; Martinaud et al., 2012), and 46 age-matched control participants. Word, object, and face processing were measured with comparable tests across different levels of processing to investigate the specificity of category-selective impairments. We present two complementary analyses of the extensive behavioural battery: (1) a data-driven analysis of the whole patient group, and (2) a single-subject case-series analysis to test for deficits and dissociations in each individual patient. This complementary approach was taken to compare whether and how these two different data-analysis approaches affect the conclusions drawn from the largest dataset of visual perceptual function after stroke currently available in order to (re-)interpret previous research into category-selectivity following stroke.

This study and the applied methods for analysis were not pre-registered. We report how we determined our sample size, all data exclusions, all inclusion/exclusion criteria, whether inclusion/exclusion criteria were established prior to data analysis, all manipulations, and all measures in the study.

## 2 Methods

### 2.1 Participants

#### 2.1.1 Stroke patients

64 patients with a single stroke in the posterior cerebral artery (ischemic or haemorrhagic) were recruited from two UK centres (University College London, University of Manchester) over a 24-month period. At the London site, patients were recruited from the PLORAS database (Seghier et al., 2016) and a specialist hemianopia clinic at the National Hospital for Neurology and Neurosurgery, University College London Hospitals. At the Manchester site, patients were recruited from local neurology clinics at Salford Royal Hospital, UK, and The Walton Centre, Liverpool, UK. All patients had had a single stroke at least 9 months prior to participation. Patients with bilateral strokes were included as long as it was highly likely that they had suffered a single episode of stroke. Only lesions affecting the cortical territory of the posterior cerebral artery were included; patients with an isolated thalamic or cerebellar stroke were excluded (see Supplementary Table 1). Patients with head injuries, or diagnosed developmental, psychiatric, or other neurological disorders were excluded.

Table 1 summarises the demographic information and background neuropsychological data. All patients were native English speakers, and mainly right handed. The laterality subgroups were not selected to be matched across demographic variables, but were not significantly different in terms of age (left vs. right: t (53) = 1.69, *p* = .10; left vs. bilateral: t (39) = 1.48, *p* = .15; right vs. bilateral: t (30) = .06, *p* = .95), education level (left vs. right: t (53) = .42, *p* = .68; left vs. bilateral: t (39) = .18, *p* = .86; right vs. bilateral: t (30) = .42, *p* = .68), time since stroke (left vs. right: t (53) = .02, *p* = .98; left vs. bilateral: t (39) = .14, *p* = .89; right vs. bilateral: t (30) = .09, *p* = .92). All patients underwent visual field and visual acuity testing (for full descriptions of tests see, Supplementary Note 1). Visual field defects were found in 60 patients (94%). 26 (41%) patients had homonymous hemianopia and 28 (44%) had quadrantanopia (lower = 13, upper = 15). Six patients had bilateral visual field deficits (9%). All patients had normal visual acuity when using their visual aids.

**Table 1:** **Participant demographics and background neuropsychological testing**. All values represent averages, with standard deviation in parentheses, with the exception of gender and handedness which represent counts.

Demographics	Control Total	Patient Total	Left	Bilateral	Right
N	**46**	**64**	32	9	23
Age	**61.5 (14.6)**	**60.9 (13.1)**	63.9 (11.6)	57.6 (10.7)	57.9 (15.2)
Gender (M/F)	**22/24**	**52/12**	26/6	8/1	18/5
Education (years)	**15.2 (1.9)**	**14.0 (2.7)**	14.0 (2.5)	13.8 (3.6)	14.3 (2.6)
Handedness (LH/Mixed/RH)	**2/2/42**	**6/1/57**	5/1/26	1/0/8	0/0/23
Time since stroke (months)		**41.9 (49.7)**	42.3 (48.0)	40.0 (28.5)	42.0 (59.4)
Lesion volume (cm^3^)		**37.0 (35.5)**	31.8 (29.9)	61.4 (37.9)	34.7 (39.2)
**Background neuropsychology**					
Geriatric Depression Scale (max 15)		**3.68 (3.54)**	3.41 (3.13)	5.00 (4.95)	3.52 (3.50)
Oxford Cognitive Screen (max 10)		**.92 (1.29)**	.84 (1.25)	1.44 (1.94)	.83 (1.03)
WAIS-IV Digit Span (Forward, max = 16)	**10.83 (2.15)**	**10.09 (2.24)**	9.94 (2.18)	11.33 (2.78)	9.83 (2.04)
WAIS-IV Digit Span (Backward, max = 14)	**7.55 (2.12)**	**6.47 (2.10)**	6.28 (2.16)	6.33 (1.66)	6.78 (2.21)
Basic RT (ms)	**397 (112)**	**600 (344)**	607 (374)	610 (369)	573 (282)

Scores in [bold] are overall group scores, [not bold] represents patient subgroup scores.

#### 2.1.2 Control participants

Patient performance was compared with 46 control participants (Table 1), unless published norms were available. Control participants were native English speakers, with no history of developmental, neurological or psychiatric disorders. Controls were either relatives of the stroke patients, or were recruited from local adult community groups across both sites. The control group was matched to the patient group in terms of age (t (108) = .24, *p* = .81), and handedness. The control group had a marginally higher number of years of education compared to the patient group (t (108) = 2.56, *p* = .012).

Stroke patients and control participants provided written informed consent in accordance with the Declaration of Helsinki. The experiment was approved by the local ethics boards (London Queen Square Research Ethics Committee, UCL; 16/EM/0348; Manchester: North West Research Ethics Committee; MREC 01/8/094).

### 2.2 Structural scanning

Structural brain imaging data were acquired in all patients and in 22 control participants. Structural scans were acquired on two 3T Phillips Achieva scanners with 32-channel head-coils and a SENSE factor of 2.5 in London and Manchester. A high-resolution T1 weighted structural scan was acquired including 260 slices covering the whole brain with TR = 8.4 ms, TE = 3.9 ms, flip angle = 8°, FOV = 240 × 191 mm^2^, resolution matrix = 256 x 206, voxels size = .9 x 1.7 × .9 mm^3^.

#### 2.2.1 Automated lesion identification procedure

**Fig. 1:**
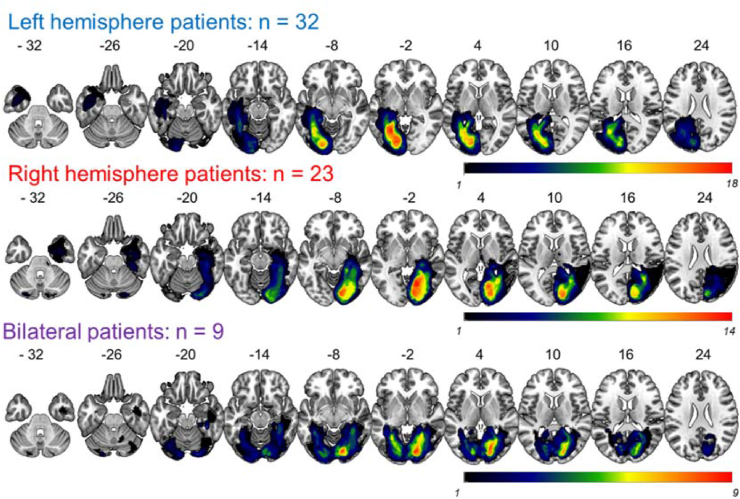
**Lesion overlap map.** Overlap of the lesion areas for each patient sub-group defined by the method described in Seghier et al. (2008). Colour bars indicate the number of patients with lesion in that area. Warmer colours = greater overlap, cooler colours = less overlap.

Automated outlines of the area affected by stroke were generated using Seghier et al.’s (2008) modified segmentation-normalisation procedure, which is designed for use with brain-injured patients and which identifies areas of lesioned tissue. Data from both the stroke patients and the control participants were subjected to the automated lesion identification procedure. Segmented images were smoothed with an 8 mm full-width half maximum Gaussian kernel and submitted to the automated lesion identification and definition modules using the default parameters. The automated method involves initial segmentation and normalising into grey matter, white matter, CSF, and an extra tissue class for the presence of a lesion. After smoothing, voxels that emerge as outliers relative to the normal population are identified and the union of these outliers generates the “fuzzy lesion map” from which the lesion outline is derived. The generated images were used to create the lesion overlap map in Fig. 1. Using this procedure, there were four patients whose lesions could not be identified. For these patients a certified neurologist (APL) manually traced the lesions using a semi-structured lesion identification technique, using the fuzzy lesion map to guide tracing.

### 2.3 Neuropsychological assessment

A detailed experimental neuropsychological battery was designed to test, systematically, a broad range of visual perceptual functions (see Supplementary Figure 2). A description of all tests included in the main analysis are included below. For a more detailed description of the full neuropsychological battery, including the development of the battery, reasons for including each test, order of test admninistration and how to access the tests and experiments see Supplementary Note 1 or Robotham et al. (2021). The majority of the battery was administered on laptops running E-Prime 2.0 software (Psychology Software Tools). The order of test administration was the same across participants and testing sites. Dependent measures in all tests were accuracy and reaction time (RT) for correct responses unless otherwise stated.

#### 2.3.1 Background tests

General cognitive function was assessed using the Oxford Cognitive Screen (OCS; Demeyere et al., 2015). Working memory was assessed using forward/backward digit span (Wechsler Adult Intelligence Scale IV-UK). Simple visual RT was measured using a basic visual task. Handedness (Edinburgh handedness inventory: short form; Veale, 2014), and mood (Geriatric Depression Scale - 15; Yesavage & Sheikh, 1986) were assessed. Three questionnaires assessed premorbid reading ability (Lefly & Pennington, 2000), post-stroke face recognition ability (Freeman et al., 2015), and post-stroke topographical orientation (Claessen et al., 2016). For more details on these tests, see Supplementary Note 1.

#### 2.3.2 Low and intermediate visual perception

To account for bottom-up effects on higher-level vision, participants underwent a series of low-level visual tests. These included measurement of visual field deficits (using the computerized Visual Field Screening test; Nordfang et al., 2019), visual acuity (FrACT Landolt C; Bach, 1996), contrast sensitivity testing (The Functional Acuity Contrast test; (Ginsburg, 1984), and colour perception (Farnsworth D-15 screening test; Linksz, 1966). Intermediate visual perception was assessed using a modified, off-line version of the Leuven Perceptual Organisation Screening Test (L-POST; Torfs et al., 2014; Vancleef et al., 2015, for details see Supplementary Note 1).

#### 2.3.3 Higher-level vision

##### 2.3.3.1 Delayed matching test & Surprise Recognition test: words, objects and faces

This test was designed to directly compare word, object and face processing within the same paradigm. The Delayed Matching part tests the ability to build a short-term representation of a stimulus and then match it with a newly presented stimulus. Participants were required to match a probe item to a target item which was either the same or novel (the target was presented at a different size to the probe image to prevent image based matching; Supplementary Figure 4a).

Following a brief intermission, a Surprise Recognition task was administered. This assessed the ability to store representations in long (er)-term memory, and again enables direct comparisons across domains. Participants were presented with pairs of stimuli, and they had to decide which of the pair (upper or lower image) they had seen before (Supplementary Figure 4b).

##### 2.3.3.2 Lexical decision

A lexical decision task was administered to assess word recognition. Participants were required to determine whether a singularly presented stimulus was a word or a non-word. Nonwords were phonologically plausible letter combinations. Items were either 3, 5, or 7 letters in length.

##### 2.3.3.3 Object decision

We assessed object decision using a version of a test described in Gerlach (2009). Participants were required to determine whether a singularly presented stimulus was a real object/animal or non-object (created by merging images of two real objects).

##### 2.3.3.4 Face familiarity

A face familiarity test was used to assess the ability to recognise a face as familiar by matching a perceived face to a representation stored in long-term memory. Participants were required to determine whether a singularly presented stimulus depicts a famous person or a novel face.

##### 2.3.3.5 Cambridge face memory test (CFMT)

The CFMT is widely used for assessing face recognition abilities and for diagnosing prosopagnosia. During this test, participants learn a set of 6 new faces and then have to recognise them amongst distractors, either in the presence of visual noise or without (Duchaine & Nakayama, 2006). The data described here excluded performance on the noise block (Corrow et al., 2018). The dependent measure was accuracy.

##### 2.3.3.6 Cambridge house memory test (CHMT)

The CHMT was used as a non-face control task for the CFMT to assess if a patient's deficit is face-specific or not. The CHMT uses the same experimental set-up as the CFMT, but involves learning a set of 6 new houses and then recognising them amongst distractors (Martinaud et al., 2012). As with the CFMT, the data on the noise block were excluded. The dependent measure was accuracy.

##### 2.3.3.7 Word reading (length: 3, 5, 7 letter words)

A word reading task was administered to measure reading RTs and word-length effects. Participants were required to read single words out-loud as quickly and as accurately as possible (items were either 3, 5, or 7 letters in length, test used by Starrfelt et al., 2009). Correct RTs from stimulus onset to vocal response were measured by a voice key and accuracy recorded by the experimenter.

##### 2.3.3.8 Word reading: lexical variables (regular, exception and non-word reading)

To further assess the type of reading deficits, a word reading test which varied lexical variables was included. Participants were required to read single words out-loud as quickly and as accurately as possible, using the paradigm described above. This test included regular and exception words and non-words selected from Graham et al. (2000) and Patterson & Hodges (1992).

##### 2.3.3.9 Text reading (NEALE)

This standardised test was used to obtain a measure of sentence reading (Neale, 1999). Participants read out-loud two passages of 26 words and 56 words, each followed by a series of comprehension questions. Participants’ responses and response time were recorded using a digital recorder. The dependent variables were words per minute and comprehension.

##### 2.3.3.10 Picture naming

Participants' naming ability was assessed using the picture naming test described in Roberts et al. (2013). Participants were required to name a series of line drawings as quickly and as accurately as possible. The same voice-key procedure as used in the word reading tests was used to record participants’ responses.

##### 2.3.3.11 Object categorisation (natural/manmade)

This task is a measure of visual object recognition without the need for a verbal (naming) output. We used a short version of the object categorisation task (Gerlach, 2001). The task requires participants to state whether singularly presented line drawing represent a man-made object or a natural object.

##### 2.3.3.12 Naming and recognition of famous faces

Famous face recognition was assessed using the face naming test described in Roberts et al. (2015). Participants were asked to name each famous face. If they could not produce the name, participants were encouraged to describe the individual as specifically as possible (e.g., why the person is famous). The dependent measure was accuracy. Scores on this test were divided into a naming score and a recognition score. Any item that was correctly named was also counted as successfully recognised. Any items which could not be named but detailed semantic information could be recalled was counted as correct recognition only. Items which could not be named and only a vague sense of familiarity but no semantic information were counted as incorrect.

### 2.4 Principal components analysis (PCA)

PCA was implemented to understand the underlying dimensions of variation in higher-level visual deficits. Table 2 shows the tests that were included in the PCA. Note that the amount of left visual field impairment was not correlated with any other task and so was excluded. A total of 3% of data were missing in the current study, either due to technical reasons or related to the severity of the patient. Missing data were imputed to improve statistical power for the PCA using probabilistic principal components analysis (PPCA) (Ilin & Raiko, 2010). PPCA requires that the number of components to be specified a-priori, so a k-fold cross validation approach was used to choose the number of components giving the lowest root mean squared error for held out cases over 1000 permutations. This was achieved using the ‘pca_compsel’ function in the PCA toolbox (http://michem.disat.unimib.it/chm/) in MATLAB. Then the missing data were imputed by the ‘ppca’ function (https://www.mathworks.com/products/matlab.html). Finally, tests with multiple measures (e.g., accuracy, RT) were reduced to one measure using a fixed-factor unrotated PCA.

**Table 2:** **List of neuropsychological tests that were included in the behavioural analyses**. All the tests below were entered into the data-driven PCA analysis. The sub-set of tests in bold were used to generate the composite scores.

Low- & intermediate level vision	Words	Objects	Faces
Right Visual Field deficit	***Delayed Matching (words)***	***Delayed Matching (objects)***	***Delayed Matching (faces)***
Visual Acuity	***Surprise Recognition (words)***	***Surprise Recognition (objects)***	***Surprise Recognition (faces)***
Leuven Perceptual Organisation Screening Test (LPOST)	***Lexical Decision***	***Object Decision***	***Face Familiarity***
	***Word Reading***	***Picture Naming***	***Famous Face Naming***
	Regular Word Reading	Cambridge House Memory Test (CHMT)	Cambridge Face Memory Test (CFMT)
	Exception Word Reading	Object Categorisation	
	NonWord Reading		
	Text Reading		

The final PCA model included data from 22 neuropsychological tests (Table 2) and 63 patients. These data were entered into a varimax-rotated PCA using SPSS (version 25). Factors with an eigenvalue >1 were extracted and orthogonal rotation applied to aid the cognitive interpretability of the principal components. The adequacy of the sample size for the PCA was determined using the Kaiser Meyer Olkin (KMO) measure.

To put the patient's performance on the visual perceptual tasks into perspective, control norms were projected into the PCA space by normalising the average control group performance on each neuropsychological test to the patient group. The factor coefficients for each neuropsychological test from the patient PCA solution were used to generate factor scores for an average control participant, which can be plotted alongside the patient data. The same procedure was followed to project in a “cut-off” score representing 2 standard deviations away from the control mean on each factor.

### 2.5 Composite score analysis

In conjunction to the data-driven analysis, a single-subject case-series analysis was conducted by deriving composite scores for the three domains of interest (words, objects, and faces). This differed from the approach above in two ways: (1) both controls and patients were included; (2) a sub-set of tasks were used to ensure task demands were matched across domains (Table 2, bold). Composite scores were generated for each domain by using unrotated fixed-factor PCA to create a single weighted average of the tests.

To assess the presence of a deficit in each domain (words, objects, faces), the performance of each individual patient was compared to the control group using single case statistics. As performance in the control group was correlated with age for all domains (words: r (46) = −.308, *p* = .04; objects: r (46) = −.541, *p* < .0001; faces: r (46) = −.398, *p* = .006), we used age as a covariate in this analysis. Education did not significantly correlate with performance in any domain and was not included as a covariate (words: r (46) = .174, *p* = .246; objects: r (46) = .181, *p* = .230; faces: r (46) = .037, *p* = .807). For this analysis we used the Bayesian test for a deficit allowing for covariates (BTD_cov) (Crawford et al., 2011) with the BDT_Cov_Raw.exe programme (https://homepages.abdn.ac.uk/j.crawford/pages/dept/SingleCaseMethodsComputerPrograms.HTM).

In addition to testing for significant deficits for each patient, we also tested for the presence of dissociations between pairs of domains (e.g., words vs. faces) for each patient. To evaluate for dissociations in the presence of variations in performance related to age, we used the Bayesian standardised difference test allowing for covariates (BSDT_cov) (Crawford et al., 2011) with the BSDT_Cov_Raw.exe programme (https://homepages.abdn.ac.uk/j.crawford/pages/dept/SingleCaseMethodsComputerPrograms.HTM). Patients have to fulfil three criteria to be classified as showing a putatively classical dissociation: (1) performance on task X must differ significantly from that of the control group, (2) performance on task Y should be within the control range, and importantly (3) the difference in performance of that patient on tasks X and Y must differ significantly from the difference scores of the control group on tasks X and Y (Crawford et al., 2011).

In addition to using unrotated, fixed-factor PCA to generate composite scores, a non-parametric approach was also taken by converting subjects scores to ranks and summing across tasks. Using the same single-case statistics approach described above revealed highly similar results (Supplementary Figure 7).

## 3 Results

### 3.1 Lesion profiles

Fig. 1 shows the lesion overlap map for all stroke patients. Lesions covered the posterior cerebral artery territory and aligned with previous descriptions of posterior cerebral artery infarcts (Martinaud et al., 2012; Phan et al., 2007). The maximal lesion overlap was in the medial occipital lobe, posterior lingual gyrus and medial posterior fusiform gyrus (Fig. 1, red). Five of the bilateral cases showed more damage in the right hemisphere compared to the left. One patient showed more damage in the left hemisphere compared to the right. Three patients showed no hemispheric differences. The bilateral group had larger lesions on average than the left hemisphere group (Table 1; t (39) = 2.48, *p* = .02). No other group differences were significant (left vs. right t (53) = .32, *p* = .75; right vs. bilateral: t (30) = 1.75, *p* = .09).

### 3.2 Group-level data-driven behavioural profiles of higher-level vision

**Fig. 2:**
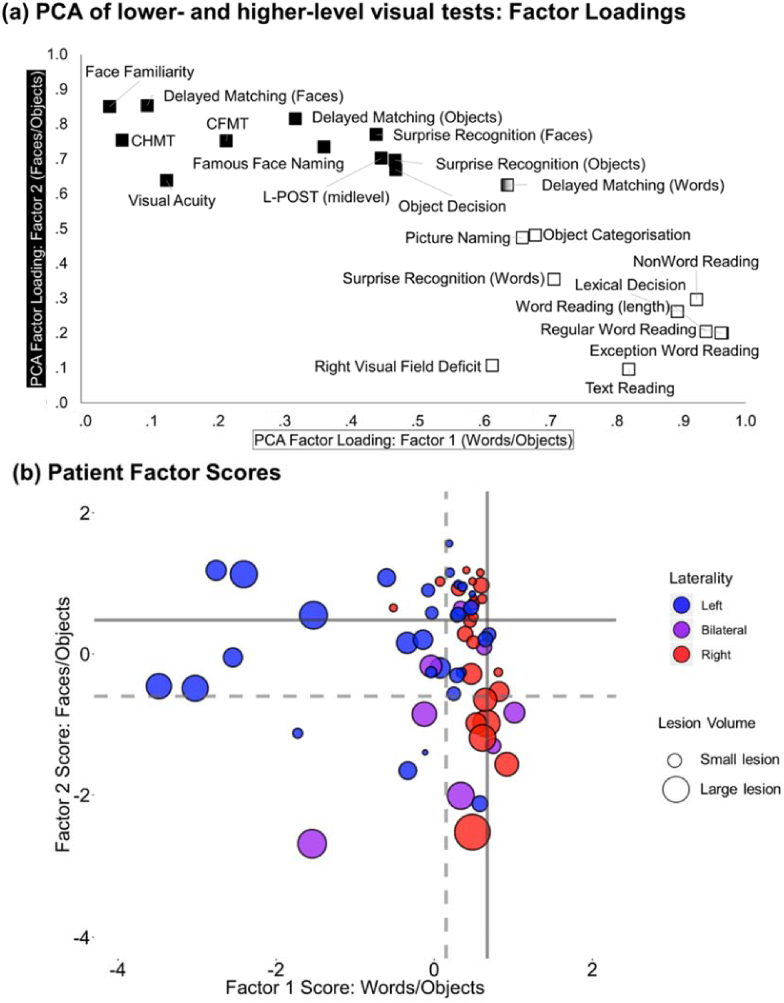
**Varimax rotated PCA of lower-level and higher-level visual tests.** (a) A two-factor solution explained 73% of the variance (KMO = .895). Each point represents the factor loading of each neuropsychological test on the two principal factors extracted from the data. Tests that load significantly on Factor 1 (Word/Object) are shown in white, tests which load significantly on Factor 2 (Face/Object) are shown in black. (b) Patient factor scores on the two principal factors extracted in the data-driven analysis. Each point represents one patient. Points are colour coded according to lesion laterality (left hemisphere strokes in blue, right hemisphere strokes in red, bilateral strokes in purple). The size of each point denotes the size of the stroke (larger points = larger stroke volume). The PCA solution was calculated on the patient group alone, thus 0 on the *x* and *y* axis represents the mean performance of the patient group. Solid lines represent the average control group performance on each factor. The dashed lines represent two standard deviations away from the control mean for each factor.

PCA using the patient data only was used to investigate the underlying structure in the behavioural dataset (Table 2). A varimax rotated PCA of the lower and higher-level visual tests produced two principal factors exceeding an eigenvalue of 1. These two factors explained 73% of the variance of the original data with a KMO of .895. Fig. 2a illustrates how each neuropsychological test loads on these two principal factors (for a description of each of the neuropsychological tests see the Methods and Supplementary Note 1).

Factor 1 accounted for 58% of the variance and contained all tests of word processing and some tests of object processing (Picture Naming, Object Categorisation). Factor 1 also included a test of low-level visual perception (severity of right visual field deficit). Performance on this factor correlated with lesion volume (r (63) = −.33, *p* = .008). This factor will be referred to as the Word/Object factor throughout the paper. Factor 2 accounted for 14% of the variance and contained all tests of face processing and some tests of object processing (Object Decision, CHMT). Factor 2 also included tests of low (visual acuity) and intermediate visual perception (L-POST; Torfs et al., 2014). Performance on this factor correlated with age (r (63) = −.40, *p* = .001), lesion volume (r (63) = −.58, *p* < .0001), and time since stroke (r (63) = −.27, *p* = .032). This factor will be referred to as the Face/Object factor throughout the paper.

This two-factor solution remained stable regardless of the data-driven analysis used. Hierarchal cluster analysis of the same data yielded identical results (Supplementary Figure 5a). The two-factor solution also remained stable regardless of which tests were included in the PCA; a varimax-rotated PCA calculated on the higher-level visual tests only, i.e., without the low-or mid-level visual tests, also revealed a two-factor solution (Supplementary Figure 6).

Fig. 2b displays the factor scores for each of the 64 patients on the two principal factors. On Factor 1 (Word/Object) there was a greater effect of laterality, with poorer performance primarily observed in patients with left hemisphere lesions. Conversely, for patient scores on Factor 2 (Face/Object) there were no clear effects of lesion laterality, despite predictions in the literature regarding larger effects on face processing following right hemisphere or bilateral damage. Fig. 2b also shows the relative performance of the control group on the two principal factors (note that because the PCA solution was calculated based on the patient data alone, the 0 point on the x/y axes represent the mean performance of the patient group). The control group showed a low degree of variability on Factor 1 (vertical dashed lines in Fig. 2b), reflecting the consistent performance across the control group on tests of word recognition. This was in contrast to the high degree of variability on Factor 2 (horizontal dashed lines in Fig. 2b).

Using the control data to create a cut-off for impaired function, the full spectrum of visual perceptual deficits post-stroke can be revealed. The majority of patients were within the control variation on both factors (18 left, 16 right, 2 bilateral). A small subset showed impairments on both factors (3 left, 2 bilateral). Generally performance was related to lesion size. Indeed, patients showing more preserved performance had smaller/more selective lesions (Fig. 2b, top right), and patients with poor performance had large (bilateral) lesions (Fig. 2b, bottom left). The remaining patients showed disproportionate performance on one factor; 12/64 patients were outside the control variation Factor 1 (Words/Objects; 10 left, 1 right, 1 bilateral), and 10/64 patients were outside the control variation on Factor 2 (Faces/Objects; 1 left, 6 right, 3 bilateral). Interestingly more patients were outside the control variation on Factor 1 (Words/Objects) than Factor 2, which aligns with previous reports that selective word recognition deficits are more common than pure face recognition deficits (Farah, 1991; Martinaud et al., 2012; Rossion, 2018).

### 3.3 A classical single-subject analysis of higher-level vision

**Fig. 3:**
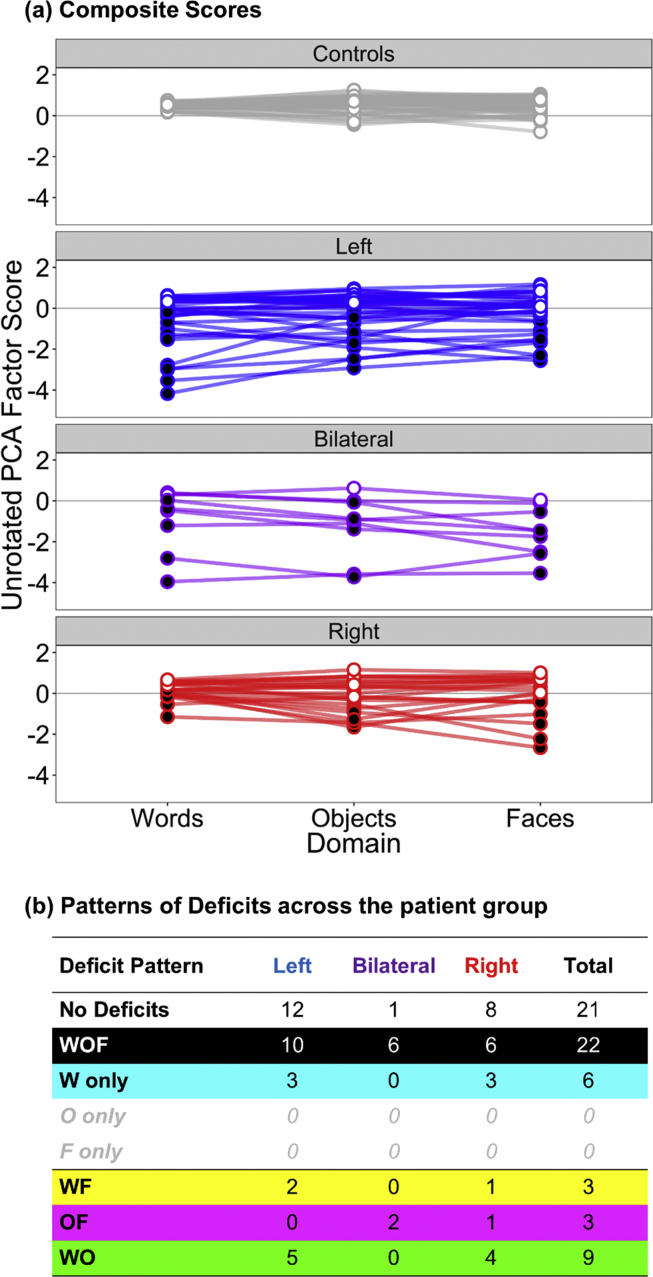
**Single-subject composite score deficit analysis.** (a) Raw composite scores for each participant sub-group for word, object and face processing. Patients showing a statistically significant deficit within a domain compared to the control group are highlighted with black circles. (b) Table summarising the pattern of significant deficits (one-tailed) across the patient group. Colours in the table correspond to the shading in Fig. 4.

The data were also analysed as a case-series, by deriving composite scores for the three domains for all subjects. Fig. 3a displays the raw composite scores for all subjects (see Supplementary Table 2 for the factor loading tables for each composite score). The control group show equivalent performance across all three domains, but show differential variability between domains. In the word domain, the control group show very little variability and greater variability in the object and face domains. Fig. 3a also highlights which patients show a significant deficit within each domain compared to the control group using the Bayesian deficit test (BTD_cov) accounting for age (Fig. 3a, black dots; Crawford et al., 2011). All three patient groups show greater variability in performance within each domain than the control group.

**Fig. 4:**
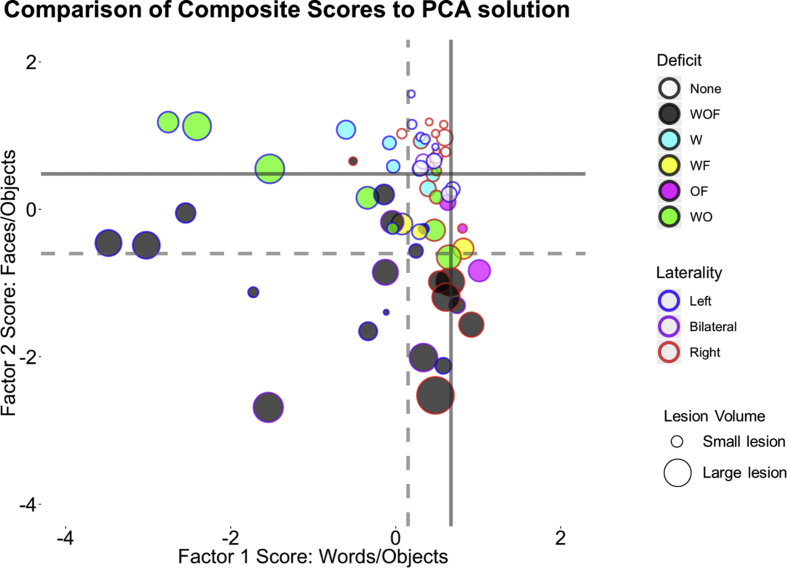
Comparison of the PCA solution described in Fig. 2b and the results of the deficit analysis in Fig. 3b. Each point represents one patient. As in Fig. 2b, the border colour of each point is colour coded according to lesion laterality (left hemisphere = blue, right hemisphere = red, bilateral = purple). The size of each point denotes the size of the stroke (larger points = larger stroke volume). The fill of each point is coloured according to the pattern of deficits described in Fig. 3b. Solid lines represent the average control group performance on each factor. Dashed lines represent two standard deviations away from the control mean.

Fig. 3b summarises the pattern of deficits across the patient group; the most common pattern was either no significant deficit in any domain (21/64 patients; Fig. 3b: white), or deficits in all three domains (22/64 patients; Fig. 3b: black). Patients showing category-selective deficits in one domain were rare (6/64 patients), and all of these patients showed a category-selective impairment for words (Fig. 3b; cyan). No patient showed a category-selective deficit for faces or objects. The remaining patients (15/64 patients) showed deficits in two domains. The most common deficit was for words and objects (Fig. 3b: green). This pattern of results is replicated when taking a non-parametric approach to creating composite scores (see Supplementary Figure 7) so it is not an artefact of the method chosen for creating the composite scores.

Patients who showed at least one significant deficit were submitted to further statistical analysis to establish the presence of significant dissociations between pairs of domains (Crawford et al., 2011). The full table of results is presented in Supplementary Table 3. From the sample of 43/64 patients who showed at least one significant deficit, 34 patients also showed evidence of a dissociation between (at least) two domains of interest. Again, the dissociations tended to reflect disproportionate deficits for word recognition compared to face and/or object recognition – 5/6 patients who showed a selective impairment for words only, also showed classical dissociations between words/faces, and words/objects, indicating a pure deficit in these patients. In contrast, only one patient with a bilateral lesion showed disproportionate deficits for faces and objects compared to words.

### 3.4 Comparing PCA solution with composite scores

The data-driven PCA and the single-subject analyses of composite scores aligned closely with one another (Fig. 4). Neither analysis highlighted consistent category-selectivity, rather objects aligned with both words and faces in the data-driven analysis, and very few patients showed category-selective deficits in the composite score analysis. Patients who showed no measurable deficit in the single-subject analysis of the composite scores also performed within the normal limits of controls on both PCA factors (Fig. 4; white circles). These patients had smaller lesions, reflecting the small behavioural impact of a selective lesion and/or the better propensity for recovery. In contrast, patients who showed significant deficits across all domains in the single-subject analysis performed poorly on both principal factors (Fig. 4; grey circles). These patients typically had larger (bilateral) lesions, perhaps reflecting general severity.

Patients who showed a word-selective deficit in the single-subject analysis (Fig. 4; cyan) fell within normal control performance on the “Face/Object” Factor 2 (which contained all the face recognition tests and some object tests), but below control performance on “Word/Object” Factor 1 (which contained all the tests of reading). These patients also had smaller lesions, and contained a mixture of left and right hemisphere patients, although the three left hemisphere patients performed relatively worse. Similarly, patients who exhibited a double deficit for words and objects in the single subject analysis (Fig. 4; green) performed poorly on Factor 1 (Words/Objects), but not on Factor 2 (Faces/Objects). However, these patients performed relatively worse on Factor 1 compared to the word-selective patients (cyan), and showed larger lesions. Again, the left hemisphere patients performed worse than the right hemisphere patients. Conversely, the patients who showed a disproportionate deficit for faces and objects (Fig. 4; magenta), performed poorly on Factor 2, but not on Factor 1. Finally, patients who showed a double deficit for words and faces (Fig. 4; yellow), fell in the middle of the graph, but aligned more closely to the generally severe patients (Fig. 4; black).

## 4 Discussion

The BoB project explored the range and specificity of visual perceptual deficits following posterior cerebral artery stroke. Single-case studies, where patients are typically recruited based on their behavioural profile, are among the strongest evidence for category-selective organisation of words and faces within the vOTC. However, when considering larger groups of patients, or considering patients recruited based on lesion location, there is growing evidence that impairments in word and face recognition are more commonly associated even following unilateral lesions (Asperud et al., 2019; Behrmann & Plaut, 2014; Gerlach et al., 2014; Martinaud et al., 2012; Roberts et al., 2013). The BoB project is unique in three key aspects: (1) patients were recruited solely on the basis of lesion location, and not behavioural profile (2) a “control” domain (i.e., object processing), was tested with the same sensitivity as the domains of interest (i.e., words and faces) to assess the true selectivity of category-selective deficits, and 3) each domain was assessed with several tests, spanning different levels of processing, rather than being indexed by one key measure. The combination of these aspects means that our sample not only included patients with category-selective deficits, but also patients with mild and severe impairments, and that their performance across domains was measured in detail. The critical finding was a spectrum of visual perceptual impairment post-stroke, both in a data-driven analysis of the whole patient group, and in a targeted single-subject case-series analysis. The most common pattern was of associations between words, objects, and faces; patients either showed deficits across all domains or in no domain. Dissociations were observed, but they were the exception and not the rule. Category-selective impairments were found in only a minority of patients, all of whom showed disproportionate deficits for words. Importantly, such selective word impairments were found following both left and right unilateral PCA stroke.

The two factors that emerged in the data-driven analysis echo Farah's (1990, 1991) suggestion that patterns of impairment in visual agnosias reflects two types of perceptual processing, rather than three category specific subsystems. Based on patterns of associations and dissociations between impairments in word, object, and face recognition, Farah suggested that face recognition relies on holistic processing and word recognition on part decomposition/featural processing, while both processes are involved in recognition of common objects. Our finding that some object recognition tasks factor in with reading tasks (Factor 1) and other object tasks with face recognition (Factor 2) could potentially be explained within this framework. We do think, however, that the relationship is more complex. While the loadings of specific tests on the two factors (Fig. 2a) are similar to what one might predict from Farah's account, the pattern of the individual patients' scores on the two factors (Figs. 2b and 4) does not follow as readily. This is particularly true if the two processing types are lateralised as suggested by Farah (e.g., 2004) so that part-based processing is left lateralised and holistic processing right lateralised. While the patients most impaired on the Factor 1 (words/objects) were left hemisphere patients with rather large lesions, which would fit with Farah's model, some patients with rather small left hemisphere strokes were impaired on both factors, and also showed across the board impairments on the composite scores in the single case analysis. Comparing Farah's (1990) review targeting patterns of reported deficits in early single case studies to our case series analysis based on matched, sensitive measures, and single case statistics (Fig. 3b and supplementary Table 3) also shows a relative lack of category selective deficits in our material. This is likely to partly reflect our recruitment based on lesion location rather than behaviour, but also perhaps that some previously reported dissociations would not come out as significant if subjected to statistical testing (see Laws, 2005; Starrfelt & Behrmann, 2011 for examples of this). An issue with earlier case studies has been the lack of direct statistical analyses of both deficits and dissociations, but also that impaired and (in particular) preserved domains have been defined based on one or a few tests. In the current analysis, both the PCA-components and the theory driven composite scores include performance on several, carefully matched tests of each domain. There may be more specific patterns of performance underlying these scores, depending on e.g., levels of processing, but the convergence of the two types of analysis indicates that the overall pattern is of association rather than dissociation across domains.

This greater commonality between word, object, and face recognition even following unilateral lesions suggests that the system underlying visual recognition is more bilaterally distributed and shared than indicated by the single case literature (Behrmann & Plaut, 2013, 2015; Roberts et al., 2013), and although category-selective patients may exist, they are rare (Barton et al., 2002; Gaillard et al., 2006; Rossion, 2018). One potential advantage of having a bilateral system underpinning any cognitive function is that the system will be somewhat more resilient to the effects of damage, because following unilateral injury to either hemisphere (a) the secondary effects associated with damage (e.g., noisy activation) are contained within the damaged hemisphere and (b) the contralateral hemisphere is able to compensate at least partially and also can help the disordered hemisphere through increased functional connectivity (Behrmann & Plaut, 2013, 2014; Jung & Lambon Ralph, 2016; Rice et al., 2018; Schapiro et al., 2013). Thus, in any bilaterally supported cognitive system, unilateral damage does lead to impaired or inefficient performance but never to the same degree as when the same amount of damage is bilaterally distributed. A similar hypothesis, highlighting the shared resources underlying face and object recognition, has been proposed in the developmental literature (Geskin & Behrmann, 2018). This bilateral, yet graded account of vOTC organisation, may also reflect a broader set of organisational principles extending along the temporal lobes (Behrmann & Plaut, 2015; Rice et al., 2015, 2018).

It should be noted that a predominantly shared bilateral system for word and face processing does not preclude graded variations in functional specialisation. In line with the well-established findings in the literature (Bub et al., 1993; Gaillard et al., 2006; Leff et al., 2006), 5/64 patients in the BoB study did show a category-selective deficit for word recognition. These category-selective deficits suggest that word recognition may rely in part on processes not critical for face recognition. This could reflect a combination of the following: differential connectivity to more distinct left-lateralised regions in the language network (Gerrits et al., 2019); the bilateral system for word recognition is more asymmetrically (left) distributed than for object and face recognition; a reliance on differential low-level image properties particularly important for word recognition (e.g., feature based processing) (Farah, 1992; Woodhead et al., 2011). Interestingly, the category-selective deficits for word-recognition in the current study did not strictly reflect lesion laterality; there were both unilateral left and right hemisphere cases that showed selective deficits for word recognition, albeit patients with left hemisphere damage showed relatively more severe deficits overall. This raises the possibility that there may be qualitatively different mechanisms underpinning word recognition impairments following left or right hemisphere damage (Gerrits et al., 2019).

In contrast to the word-selective deficits shown in a minority of patients, no patient showed a face-selective deficit. This is despite compelling evidence of face category-selectivity, both in healthy participants (Kanwisher, 2010), following brain damage (Barton et al., 2002; Rossion et al., 2003; Susilo et al., 2015), and following brain-stimulation in patient populations (Jonas et al., 2016; Parvizi et al., 2012). However, it is also difficult to deny the overlap between faces and other domains in larger proportions of the acquired and developmental populations (Asperud et al., 2019; Behrmann & Plaut, 2014; Geskin & Behrmann, 2018; Roberts et al., 2013). One potential reason for the lack of face-selective deficits in this sample is that because of the similarities between face and object recognition (e.g., similar low-level visual properties, similar demands on memory encoding, storage and retrieval); both domains rely on a shared cognitive/neural system, while word recognition stands apart in many of these characteristics. This, combined with our careful matching of sensitive tests across domains may have made dissociating faces and objects less likely than when comparing different test paradigms for faces and objects. Another possibility is that the lesion coverage here may miss critical regions for normal face recognition. The lateral fusiform gyrus has been shown to be critical for normal face recognition (Jonas et al., 2015; Rossion et al., 2003; Sergent et al., 1992), and whilst this region is affected in our sample (including in the patient who showed the most disproportionate performance for face recognition), it is not damaged consistently or selectively in any patient. One possibility is that the lateral fusiform gyrus falls within the watershed territory between the posterior and middle cerebral territories (Newton & Potts, 1974). This may explain why pure face recognition impairments are rare following stroke, and more often reported following other types of brain-injury (Barton et al., 2002; Rossion et al., 2003).

A third strength of the BoB project is the large control group who underwent the same sensitive testing as the stroke patients. In neuropsychological studies, control groups are often small, which restricts the estimation of natural/normal variability expected on a task, and the relationship across tasks. Therefore, a larger control group allows a better estimation of this variability, which is particularly important for establishing the presence of dissociations (Crawford et al., 2011; Gerlach et al., 2014, 2018). A key finding was that the control group's variability on face/object processing tests was not comparable to the minimal variability shown on tests of word recognition. Single word reading for literate adults is a trivially easy task, which results in high accuracy and quick/consistent RTs. When testing word reading in patient groups, deficits are often identified based on RT. Therefore, if the control group are highly consistent in responding, then only a small deviation (slowing) from the control mean will be required to classify a deficit (Patterson & Lambon Ralph, 1999). In contrast, on face/object recognition tests, performance in the control group was more variable, and therefore deficits were more evident in accuracy. Despite matching tests across domains (Supplementary Note 1), there is still the possibility that intrinsic differences between stimuli from different categories inescapably change task demands even when the experimental task is the same. Thus, it remains a challenge in the field to disentangle task-effects from category-effects (Robotham & Starrfelt, 2017a, 2017b). One potential way to equate the variability in word and face recognition may be to use more complex word recognition tests in order to induce errors or prolong reaction times in control participants, but this could change the task in other, non-trivial ways.

A final strength of this study was the broad range of perceptual deficits shown in the patient group. One potentially surprising finding was that almost one third of the patients showed no measurable deficits in any domain, and another third showed deficits across all domains. This finding reflects clinical observations of patients with mild or generally severe deficits, following minor strokes or larger strokes, respectively. This is an important issue, because whilst patients with mild or generally severe deficits are reported in the literature, they are rarely studied in the same level of detail (or in direct comparison to) patients with category-selective deficits. Therefore, the inclusion criteria used here to include patients based on lesion location, rather than behavioural profile, was done to characterise the spectrum of possible deficits. A common critique of neuropsychological patient studies is the lack of a patient control group, as it is common for any brain injury to affect at least reaction times. The data in the BoB project shows that not just any lesion in the PCA territory will lead to perceptual deficits or slowed response times, but rather that the localisation and size of the lesion is key.

The current study is an example of how cognitive neuropsychology has come to include not only single case-studies but also studies including multiple patients and patient groups (e.g., Butler et al., 2014; Ingram et al., 2020; Woollams et al., 2007). The big-data case-series approach taken in the BoB project and similar projects does not imply that classical single-subject investigations should be abandoned or ignored, rather it has allowed us, for the first time, to put patients with rare, selective deficits in their full clinical context. This approach also takes into account factors such as general severity and individual differences which are, by definition impossible to map with single cases (Ralph et al., 2002).

Our investigation relates to the debate around dissociations and associations in neuropsychology and cognitive neuroscience more generally (many of the issues pertain to functional neuroimaging as much as they do to neuropsychological data). This is a fundamental theoretical issue that goes beyond what we can fully explore in the current paper. Thus, we limit ourselves to a few key observations. There is no doubt that the rise of theory-shaping cognitive neuropsychology (from the 1970s) was founded upon the power of observed dissociations reported in elegant single-case studies. Many of these cases were so powerful that they over-turned firmly held theories of the day (e.g., the dissociation of short-term and long term memory, the dissociations of reading pathways implicated by surface and deep dyslexia (Coltheart et al., 1980; Shallice, 1988)). At the same time, some of the limitations of associations between deficits were expounded; including the fact that if two distinct functions happened to be neuroanatomical neighbours then brain damage is likely to cause dual deficits even though there is no mechanistic relationship between them. Such arguments remain a logical truism and -in their most extended form-become impossible to falsify, given the current technological limitations on spatial resolution of brain function.

Whilst dissociations and single-case studies remain a key feature of contemporary study, a growing number of investigations have noted that: (a) such dissociations need to be treated with some caution; and, (b) associations have a central role when we shift towards considering potential computational/mechanistic accounts of cognitive function. Thus, for example, Patterson and colleagues have noted the potential hazards of the “one black swan” argument in neuropsychology (e.g., Patterson et al., 2006; Woollams et al., 2007) which prefigures the influence that premorbid individual differences and non-random sampling might have on the occurrence of patient profiles and dissociations (Woollams et al., 2017). These observations motivated the simultaneous single-case and case-series analyses presented in the current paper: i.e., for the first time, to place identified single-case dissociations into the broader distributions of similar types of patient. It is entirely possible for such analyses to consolidate the presence of very robust, coherent dissociations that were first shown in famous individual case studies (e.g., ‘category-specific’ semantic impairments for animate kinds in HSVE but not semantic dementia patients (Lambon Ralph et al., 2007 after Warrington & Shallice, 1984). In short, there is no doubt that dissociations can provide uniquely powerful theoretical leverage, especially when they are replicated and placed into the broader context provided by understanding their position in the patient distribution.

The potential limitations of associations (e.g., ‘collateral damage’) are well known and often rehearsed. We note, however, a tension here for our science. If we move beyond observational science towards proposing and testing mechanistic/computational hypotheses then exploring the strength and consistency of associations becomes a central pursuit. It is inescapable that any proposed mechanism will lead to a predicted association. A sophisticated example comes from models of reading and central alexias. The well-known “triangle” model of reading (Plaut et al., 1996; Seidenberg & McClelland, 1989) led to the mechanistic proposal that word meaning (semantics) makes an important contribution to reading aloud such that with diminished influence patients should become surface dyslexic–i.e., as soon as one moves to a computational account then predicted associations will arise. This example is a useful one in that a handful of counter cases (dissociations) have been reported–thus returning to the issue of how to weigh the relative importance of dissociations over associations. A critical step, therefore, came with the Woollams et al. (2007) study which mapped reading and semantics in a very large, cross-sectional and longitudinal sampling of patients with semantic dementia patients–thus allowing one to observe for the first time the strength of the association (high) and the occurrence of a small number of dissociation cases in the broader distribution. Having done so, the study was able to consider the importance of individual differences in reading development on subsequent neuropsychological profiles, which was embodied in extensions of the computational model and later tested through independent fMRI and TMS studies (Hoffman et al., 2015; Woollams et al., 2017).

In closing, as our science increasingly strives to add mechanistic accounts to descriptive levels of analysis, then we need to consider how best to make use of the benefits and weaknesses of both dissociations and associations. The current study offers a new approach by which all patients are studied at the individual level (like any classical single case study) but are placed into the distributional context formed by patients of a very similar type, with the multidimensional distribution extracted by data-driven analytics.

## Funding

This work was funded by the Independent Research Fund Denmark [Sapere Aude to RS; DFF - 4180-00201] and supported by a programme grant and intramural funding to MALR from the 10.13039/501100000265Medical Research Council [MR/R023883/1; MC_UU_00005/18].

## Data accessibility

The conditions of our ethics approval do not permit public archiving of anonymised study data. Readers seeking access to the data should contact the corresponding author Prof. Randi Starrfelt (randi.starrfelt@psy.ku.dk). Access will be granted to named individuals in accordance with ethical and data sharing procedures governing the reuse of sensitive data, and a formal data sharing agreement approved by legal consultants at University of Copenhagen must be signed by both parties. Requestors must have the necessary infrastructure to receive and store the data securely. Legal copyright restrictions prevent public archiving of many the various instruments and test batteries used in this study, or the code used to present them. A full list of tests and experiments described in this manuscript and supplementary material is available in Supplementary Note 1. This list includes information about original references for each test and experiment and how to access them, either directly or by email to a specified co-author of this paper.

## Credit author statement

Grace Rice: Investigation, Formal analysis, Visualization, Writing – original draft.

Sheila Kerry: Investigation, Formal analysis, Writing – review and editing.

Ro Robotham: Methodology, Investigation, Project administration, Writing – review and editing.

Alex P. Leff: Conceptualization, Methodology, Supervision, Writing - review and editing.

Matt A. Lambon Ralph: Conceptualization, Methodology, Supervision, Writing - review and editing.

Randi Starrfelt: Conceptualization, Supervision, Methodology, Data curation, Funding acquisition, Project administration, Writing – original draft.

## Declaration of competing interest

The authors declare no competing interests.
