# Supplementary material for: Category-selective deficits are the exception and not the rule: Evidence from a case-series of 64 patients with ventral occipito-temporal cortex damage
Source: Cortex. 2021 May;138:266–81. doi: 10.1016/j.cortex.2021.01.021 (PMC8064027; doi:10.1016/j.cortex.2021.01.021)
Supplement: Multimedia component 1 [file mmc1.pdf]

Supplementary Materials

These materials accompany the paper published in *Cortex*;

Category-selective deficits are the exception and not the rule: Evidence from a case-series of 64 patients with ventral occipito-temporal cortex damage

Grace E Rice[1], Sheila J Kerry[2], Ro J Robotham[3], Alex P Leff[2], Matthew A Lambon Ralph[1], & Randi Starrfelt[3]*

1. MRC Cognition and Brain Sciences Unit (CBU), University of Cambridge, UK
2. University College London Queen Square Institute of Neurology, UK
3. Department of Psychology, University of Copenhagen, Denmark

* Corresponding author Randi Starrfelt (randi.starrfelt@psy.ku.dk)

Contents

Supplementary Table 1: Patient recruitment breakdown 2
Supplementary Note 1: The Back of the Brain project protocol 3
Designing the Back of the Brain project test battery 3
The Back of the Brain Project: Behavioural test battery 5
Background information 5
Low-level and intermediate visual perception 8
High-level visual processing 10
Associated functions 16
Full list of tests included in the Back of the Brain project and how to access them 18
Supplementary Note 2: Stability of the PCA solution 21
Hierarchal cluster analysis of the tests of lower and higher-level visual processing 22
PCA of higher-order visual tests 23
Supplementary Note 3: Composite Score Analysis 26
Composite Score Factor Loading Tables 26
Dissociation Analysis 26
Non-parametric equivalent of the Composite Score Analysis 27
References 30

Supplementary Table 1: Patient recruitment breakdown

	**Manchester**	**London**
Identified (posterior cerebral artery stroke + provisional BoB criteria)	109	91
Not meeting inclusion criteria	24	12
Declined participation	6	17
Unable to contact	51	15
Completed testing but excluded from analysis	4	3
Additional lesion outside posterior cerebral artery	*2*	*0*
Posterior cerebral artery without cortical involvement	*2*	*1*
Did not complete testing	0	*2*
**Recruited & Completed Testing**	**23**	**41**

Supplementary Note 1: The Back of the Brain project protocol

Designing the Back of the Brain project test battery

The “Back of the Brain (BoB)” behavioural test battery was designed to test the range and specificity of visual perceptual deficits following stroke. The test battery had the following constraints determined by the project protocol and budget:

A. Maximum 9 hours completion time for a typical patient with brain injury.
B. Assessment of each patient spread over maximum three sessions distributed over maximum three days.

Creating the test battery involved the following steps:

Step 1: Identify lower-level, intermediate and higher-level visual perceptual functions as well associated functions that are relevant for the study:

A literature search was carried out to identify functions that could be relevant to assess (summarised in Supplementary Figure 1).

Supplementary Figure 1: Functions that could be relevant to assess in the BoB project.

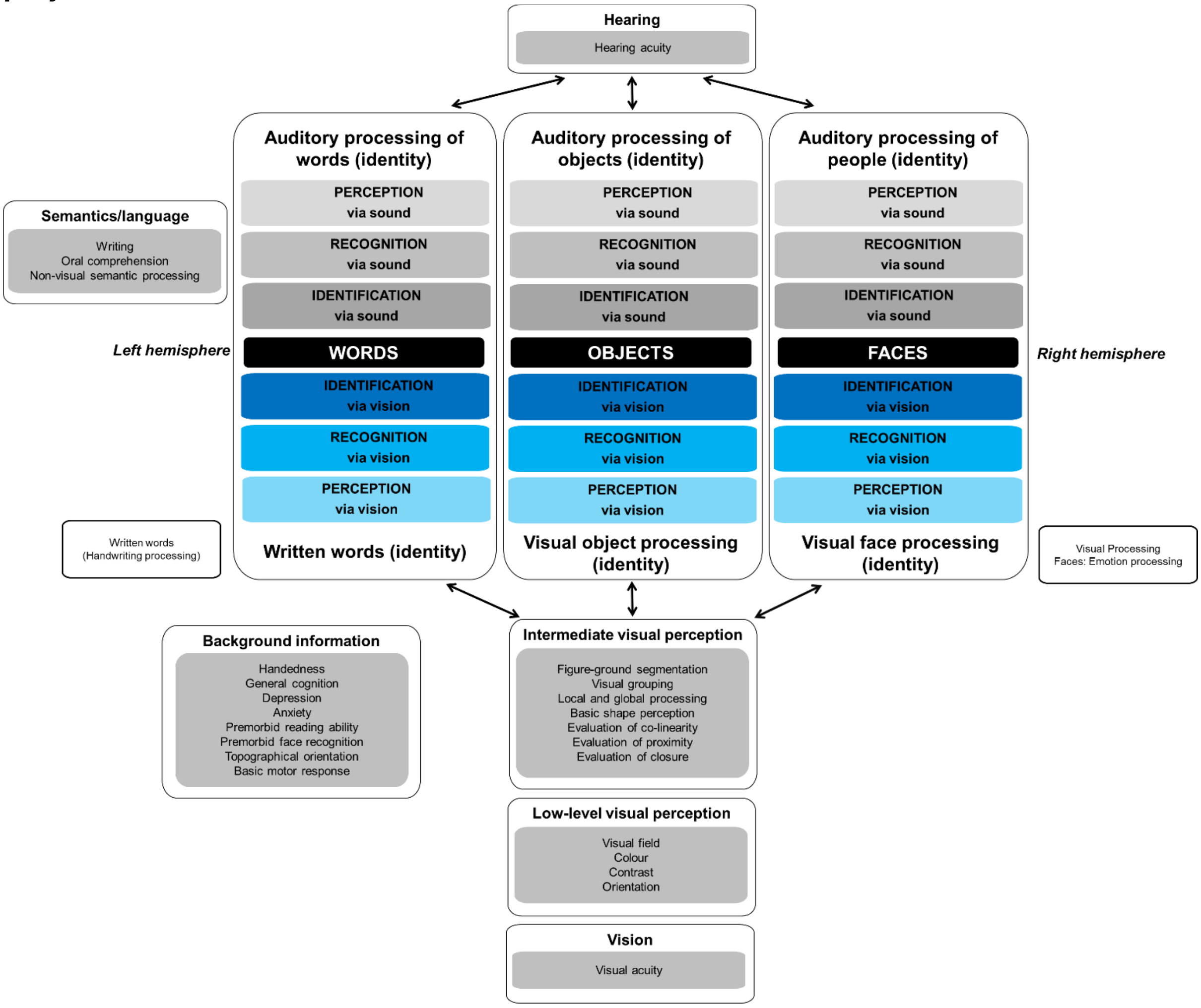

Step 2: Literature search to identify relevant tests and create a “dream scenario” test battery.

Tests that fulfilled as many of the following criteria as possible were prioritised:

(a) Available in English
(b) Short testing time to limit fatigue
(c) Validated and/or previously used in research
(d) Central/vertical presentation of stimuli to limit the effects of hemianopia on performance
(e) Tests assessing non-visual functions must be as visually simple as possible

This led to a “Dream scenario” test battery that was piloted in a group of patients with stroke.

Step 3: Prioritise functions to create a “final version” of the test battery.

After piloting the “dream scenario”, many tests/experiments were shortened or removed from the test battery to fit the time constraints of the project. Only those considered most important were included in the final test battery (Supplementary Figure 2).

Supplementary Figure 2: Final BoB test battery. Tests in bold were included in one of the two main analysis presented in the main text.

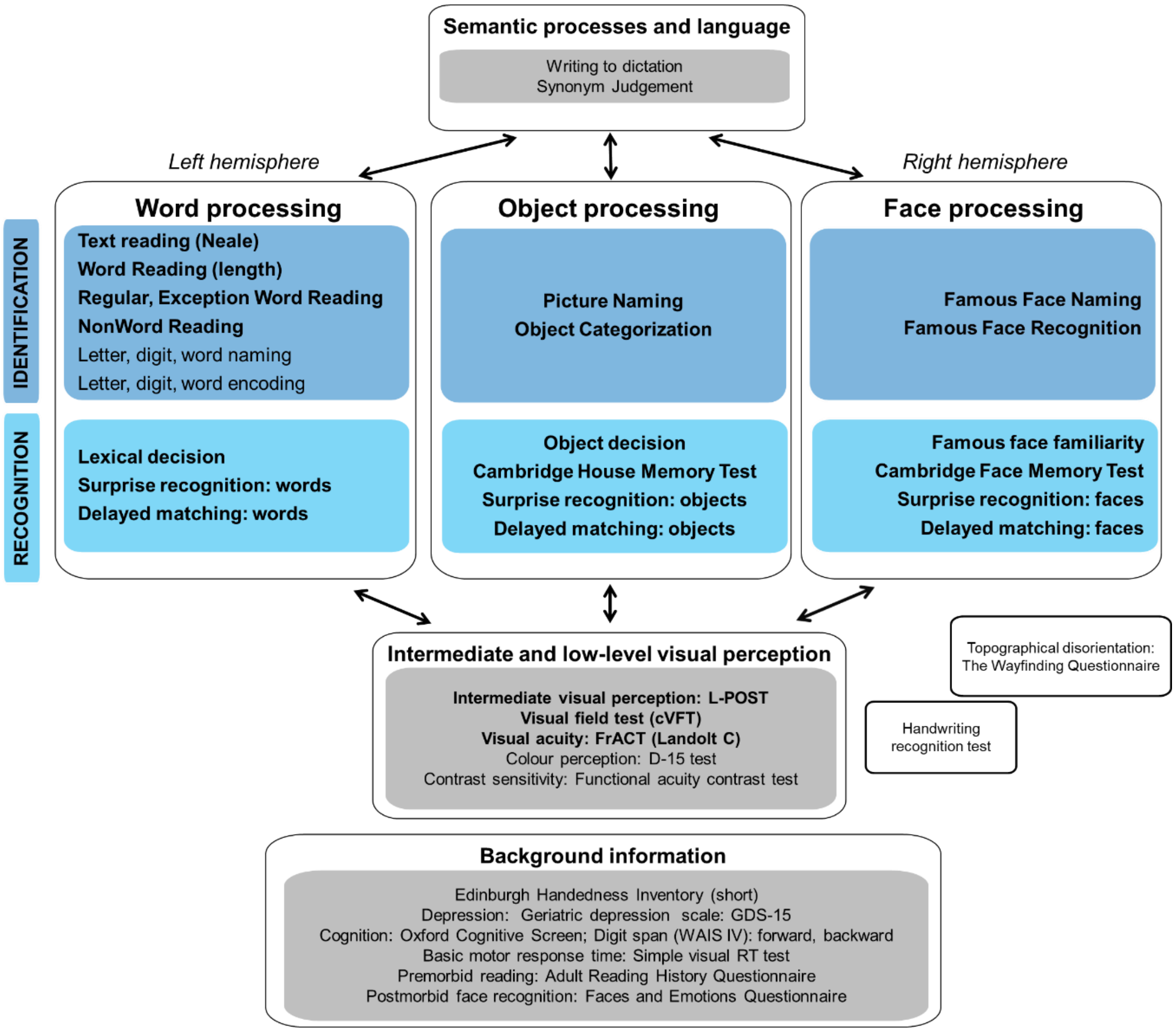

The Back of the Brain Project: Behavioural test battery

Tests were either carried out using paper-and-pencil, Laptop computers with a screen resolution of 1366 x 768 (London: Dell latitude e6430 running on CORE i5 Windows 7 Professional; Manchester: Lenovo T560 running on Windows 7), or desktop computers with a screen resolution of 1920x1080 (Windows 7 Enterprise, 64-bit operating system, 24 Inch BenQ XL2430T screen). The order of test administration was consistent across both testing sites.

Further information for each of the tests included in the battery is provided below. A list of the included tests with original references as well as information on accessibility and how the tests can be acquired is presented following this description. Tests are categorised into background tests, tests of lower-level vision, and tests of higher-level vision.

Background information

Edinburgh Handedness Inventory - Short Form

Reason for inclusion: A questionnaire related to handedness was included as some of the core hypotheses of the BoB project are related to cerebral lateralisation, which is known to be linked to handedness.

About the test: The Edinburgh Handedness inventory (Oldfield, 1971) is the most commonly used handedness questionnaire. The original questionnaire includes 10 items. In the BoB project a shorter 4-item version was used. The 4-item version is developed based on confirmatory factor analysis that was shown to have good reliability, factor score determinacy, and has been shown to correlate with scores on the 10-item inventory (Veale, 2014).

Geriatric Depression Scale - 15 (GDS-15)

Reason for inclusion: A depression screening tool was included as depression is known to be common amongst stroke survivors and known to affect performance on cognitive tests (Hackett et al., 2005).

About the test: The Geriatric Depression Scale (GDS) is a self-report measure that was designed for depression screening in older adults (Yesavage et al., 1983). One of the main advantages of the tool is that questions are answered with simple yes/no options making it easier for participants with cognitive challenges to complete. The questionnaire originally included 30 items, but a shorter 15 item version, the GDS-15, has been shown to have similar test properties as the longer version (Yesavage & Sheikh, 1986). As most subjects in the BoB project are in the older age range, the GDS was chosen as a depression screening tool. The shorter version of the tool was chosen to limit assessment time.

Oxford Cognitive Screen (OCS)

Reason for inclusion: Stroke can lead to a wide range of cognitive deficits. Screening of cognitive deficits was carried out to determine whether participants have substantial cognitive deficits (in other domains than visual perception), in order to enable interpretation of poor performances on the experimental tasks included in BoB. Amongst others, memory, language, executive deficits, and neglect could potentially affect performance on many of the

experiments. Although OCS is not a dementia screening tool, it should also enable identification of participants with severe cognitive deficits, who may have dementia.

About the test: The OCS is a cognitive screening tool specifically designed for stroke patients (Demeyere et al., 2015). It covers the main cognitive domains that are commonly affected by stroke: Language, Attention, Memory, Praxis, and Number processing. OCS sub-tests: Picture naming; Semantics/picture pointing; Orientation; Visual field; Sentence reading; Number processing (writing and mental arithmetic); Broken hearts (neglect); Meaningless gesture imitation (praxis); Memory (recall & recognition); Trail tasks (executive functions).

Digit span: forwards and backwards

Reason for inclusion: As working memory is not assessed in the Oxford Cognitive Screen, a measure of forwards and backwards digit span was included in the test battery. Many of the experiments included in the BoB put substantial demands on working memory. Few patients in the project are expected to have aphasic deficits, so verbal assessment of working memory was considered appropriate.

About the test: Digit span (forwards and backwards) from the Wechsler Adult Intelligence Scale IV (Wechsler, 2010) was chosen as it enables fast and efficient assessment of working memory and has detailed normative data available.

Basic motor response

Reason for inclusion: Stroke can lead to general cognitive slowing. A basic visual task was included to provide a baseline lower level visual reaction time measurement that can enable interpretation of reaction time data acquired in more complex experimental tests.

About the test: This experimental task was specifically designed to measure basic reaction times in the BoB project. Many of the reaction time tasks included in BoB project are 2AFC tasks, therefore a simple visual 2AFC task was designed in which participants must determine whether a stimulus, a narrow black rectangle that extends from one side of the screen to the other, is presented at the top or bottom of the screen (with a grey background). The stimulus was chosen to ensure that patients with hemianopia and various forms for agnosia could complete the task. The test includes 4 practice trials and 20 test trials. The dependent measure was correct reaction times.

Adult Reading History Questionnaire

Reason for inclusion: Some of the more specific questions of the BoB project involve relations between pre-stroke reading experience/proficiency and severity of post-stroke alexia and prosopagnosia. Therefore, an assessment tool for reading abilities prior to stroke was included. In addition, identification of patients with dyslexic reading is important when interpreting data from reading tests.

About the questionnaire: While there are many questionnaires designed to identify dyslexia in children, there are only a limited number of questionnaires designed to assess reading difficulties in adults. The Adult Reading Questionnaire (Lefly & Pennington, 2000) is designed to retrospectively assess developmental reading problems. It includes questions about experience with reading during childhood and reading ability in adulthood.

Wayfinding Questionnaire

Reason for inclusion: Wayfinding problems and topographic agnosia / amnesia have been reported following posterior cerebral artery stroke, and the literature suggests that many patients with acquired prosopagnosia also have wayfinding problems. While this function is not experimentally tested in the BoB-battery, questions about wayfinding abilities were included.

About the questionnaire: The Wayfinding Questionnaire is designed to identify wayfinding problems after stroke (Claessen et al., 2016). The following four questions from the original wayfinding questionnaire were included in the final protocol. The participants are asked to provide responses related to their current post-stroke abilities. Response options follow a Likert scale from 1 (Not at all applicable to me) to 7 (fully applicable to me):

- I am good at understanding and following route descriptions.
- I can always orient myself quickly and correctly when I am in an unknown environment.
- I can easily find the shortest route to a known destination.
- I can usually recall a new route after I have walked it once.

The following two questions were also included:

- Since my stroke/head injury it is more difficult for me to find my way and orientate myself (also Likert scale)
- Is there anything else you want to tell me about your navigation and way finding abilities before your stroke? (Open question)

Face recognition questionnaire

Reason for inclusion: A face questionnaire was included to enables identification of patients with prosopagnosia. Indeed, one of the common core criteria used to diagnose prosopagnosia is that a patient has "complaints of impaired face recognition in daily life”. Another reason for including a face recognition questionnaire is to enable the analysis of correlation between self-reporting of face recognition difficulties and performance on face processing tasks.

About the test: The Faces and Emotions questionnaire was designed to evaluate self-reported face recognition deficits (Freeman et al., 2015). The questionnaire is freely available. The following 10 questions were selected from the Face Identity Recognition part of the questionnaire. Questions were adjusted to be appropriate for people with acquired brain injury, and patients were asked to report their experiences following their stroke. Response options follow a scale from 1 (Definitely agree) to 4 (Definitely disagree), participants responded out-loud and their response was noted by the experimenter:

- I can usually remember what someone’s face looks like, even if I’ve only met them once.
- I find it difficult to decide whether I know a face or not.
- I have trouble finding my friends in a crowded room.
- I occasionally fail to recognise myself in old photos.
- I often have conversations with people who appear to know me, but (at least initially) I have no idea who they were.
- I often rely on distinctive bodily features, hair, or clothing to help identify people.
- I rarely confuse characters in TV programs.

- I usually recognise my friends in old photographs.
- If I saw my neighbour at the shops, I would recognise them.
- If a friend changed their hairstyle I would most likely be able to identify them.

To ensure that potential problems are indeed related to brain injury, two additional questions were added:

- My ability to recognise faces has got worse since my stroke/head injury (Same scale as questions above)
- Is there anything else you want to tell me about your ability to recognise faces before your stroke/head injury? (Open question)

Low-level and intermediate visual perception

Computerised Visual Field Test

Reason for inclusion: A visual field test was included in the BoB protocol as visual field defects are common following posterior cerebral artery stroke and can impact performance on many visual perceptual tasks.

About the test: The computerized Visual Field Screening Test (c-VFT) was developed at the Department of Psychology at University of Copenhagen (Nordfang et al., 2019). Commonly used perimetry tests are time consuming and can be difficult to run on patients with mobility limitations. The c-VFT can be run on a desktop or laptop computer and can therefore be carried out at bedside and only takes approximately five minutes to administer. The test probes 48 points within a radius of 10 degrees of visual angle (dva) around a central fixation cross. The points are equally sized dark circles presented against a light grey background. The stimuli probe at 1, 2, 5, and 10 degrees of visual angle. The test includes assessment of points along the horizontal and vertical meridians. Integrity of visual field along the horizontal meridian is particularly relevant for reading. The c-VFT has been validated against the Esterman test and the Humphrey Visual Field Analyzer (HFA), central 10-2 that are perimetry tests commonly used in clinical settings (Nordfang et al., 2019).

Freiburg Visual Acuity Test And Contrast Test (FrACT; Version 3.9.3)

FrACT: Landolt C Acuity Test

Reason for inclusion: The FrACT test was included to evaluate the status of participants' visual acuity when using glasses/lenses. Stroke patients are often in the older age range and many are expected to have acuity problems. Low visual acuity can affect performance on many visual tasks.

About the test: The FrACT Landolt C visual acuity test was chosen for the BoB project as, in contrast to Log MAR charts in which all stimuli are presented simultaneously on a chart, stimuli are presented one at a time in the centre of the screen. This is useful for patients with visual field deficits or who are visually disorientated. Another advantage of FrACT is that Landolt Cs are used as stimuli rather than letters. The test is therefore better suited for patients with some forms of reading deficits than tests using letters from the alphabet. The FrACT presents Landolt Cs one at a time on a computer screen with varying sizes to assess

visual acuity. It uses an adaptive staircase procedure to measure acuity threshold (Bach, 1996). For more information about the test: https://michaelbach.de/fract.

FACT: Contrast Sensitivity Test

Reason for inclusion: A contrast sensitivity test was included in the protocol. It has long been hypothesised that the visual processing of faces and words could rely differentially on low or high contrast information, and that differences in abilities to process low or high contrast visual information may explain why patients are more impaired in one category than the other. The test used here has previously been used in a large-scale study of posterior brain injury (Roberts et al., 2013).

About the test: The functional acuity contrast test (FACT; http://www.stereooptical.com/) is a diagnostic tool used to evaluate real-world vision capabilities. The test evaluates sensitivity across a range of spatial frequencies and contrast. The test comprises a progression of high-quality, sine-wave gratings that probe sensitivity to 1.5, 3, 6, 12, and 18 cycles per degree. The contrast step between each grating patch is 0.15 log units. The contrast range spans the variation of contrast sensitivity found in the normal population. Following the standard instructions, participant were asked to decide whether each grating was tilted right, or left. Normative limits which include 90% of the normal population are used to help minimize the potential for false positives. This test was done with both eyes open, rather than for each eye individually.

The Farnsworth D-15 test of colour perception

Reason for inclusion: The Farnsworth D-15 test of colour perception test was included to identify congenital colour blindness as well as acquired achromatopsia. It is possible that a deficit in colour perception affects the ability to recognise some types of complex stimuli more than others. Also it has been shown that achromatopsia can co-occur with prosopagnosia, however, little is known about the relationship between the two deficits (Bouvier & Engel, 2006)

About the test: The test is a modification of the Farnsworth-Munsell 100 Hue test (Farnsworth, 1943). The 15 cap version is intended for screening purposes (Linksz, 1966). The test contains 15 caps with different colours. The “pilot” cap is fixed to the left of the tray. The other caps are presented to the participant in mixed order. Participants are asked to “select the cap which is the closest possible match to the pilot cap”. The chosen cap is placed to the right of the pilot cap. The participant must then “choose the closest colour match to the cap that was just chosen”. This procedure is repeated until all caps have been placed in a row. Different result patterns indicate different forms of colour vision defects.

Leuven Perceptual Organisation Screening test (L-POST, midlevel vision)

Reason for inclusion: The L-POST was included to measure mid-level perceptual processing. Difficulties in processing complex visual stimuli can in some cases be caused by deficits in mid-level visual perceptual processing. By assessing mid-level visual perception, we can investigate whether some types of mid-level perceptual deficits affect the processing of some visual categories more than others.

About the test: The L-POST is a screening tool designed to assess deficits in mid-level vision (Torfs et al., 2014; Vancleef et al., 2015). An opnline version of the test is openly available here: http://www.gestaltrevision.be/tests/ It includes 15 subtests assessing a wide

range of mid-level processes, such as figure-ground segmentation, local and global processing, shape perception, and the ability to use a variety of grouping cues e.g., proximity, and closure. It is an internet based tool, designed for use in both clinical and research settings. In the original version, performance is determined on the basis of accuracy alone. For the BoB project, a modified version of the test was created in OpenSesame to enable both accuracy and reaction time measurements. In the sub-tests that use static images, the overall set-up is similar to the original version of the L-POST. Participants are presented with a target image at the top of the screen and three test images below. Participants must determine as fast as possible which of the test images is most similar to the target image. They can respond as soon as the stimuli appear on the screen, and in contrast to the original version of the L-POST in which response time is unlimited, participants in the OpenSesame version of the L-POST have a maximum of 10 seconds to provide a response. In the video sub-tests of the OpenSesame version of the test, a video is played for 5 seconds, after which the video is replaced with a question mark prompting a response that must be provided within maximum 10 seconds. This stands in contrast to the original version of the L-POST in which participants are required to provide a response while the video is running on the screen. The following sub-tests were included in the BoB protocol: Fine shape discrimination; Shape ratio discrimination (Efron); RFP contour integration; Figure–ground segmentation; Embedded figure detection; RFP texture segmentation; Kinetic object segmentation; Dot counting; Global motion detection.

High-level visual processing

Delayed Matching and Surprise Recognition test (Words, Objects and Faces)

Reason for inclusion: This test was included in order to compare recognition abilities across the categories of faces, words and objects. The experiment uses the same paradigm to assess face, word and object recognition, leading to easier comparison across categories.

About the test: *The Delayed Matching test and Surprise Recognition Test* was developed specifically for the BoB project and involves two parts: *a delayed matching test* and a *surprise recognition test* (Robotham, 2019). The *Delayed Matching* Test assesses the ability to build a short-term representation of a stimulus and then match it with the same or a novel stimulus. The *Surprise Recognition* test that is administered directly after is an old/new recognition paradigm that assesses whether participants later can recognise stimuli that were used in the *Delayed Matching* part of the test. Processing of words, objects and faces are assessed independently in each part. With its two separate parts, a distinction can be made between recognition problems that are caused by a deficit in storing a representation over longer time from deficits related to problems in creating a short-term representation of a stimulus and matching it with a currently viewed stimulus.

Delayed Matching Test

Materials: For each category, four groups of three visually similar stimuli are used (12 uncropped faces, 12 words, and 12 objects; Supplementary Figure 3a). All images are in black and white. The faces were selected from the Radboud Faces Database (Langner et al., 2010). All faces are presented in frontal view with neutral emotional expressions. Two clusters of three male faces and two clusters of three female faces are used. The three faces in a cluster have similar hairstyles and similar visual features (see Supplementary

Figure 3a). For the word stimuli, four clusters of three 4-letter words are used. Words in the same group only differ by one letter. In group 1, the first letter changes, in group 2, the second letter changes; in group 3, the third letter changes; and in group 4, the fourth letter changes. The task can therefore not be performed by focusing on a single letter position. Words are presented in lowercase writing in Arial font. The object stimuli includes four clusters of images representing four different object categories: cars, butterflies, boots, and flowers.

Supplementary Figure 3: Stimuli for the Delayed Matching Test (a) and the Surprise Recognition Test (b).

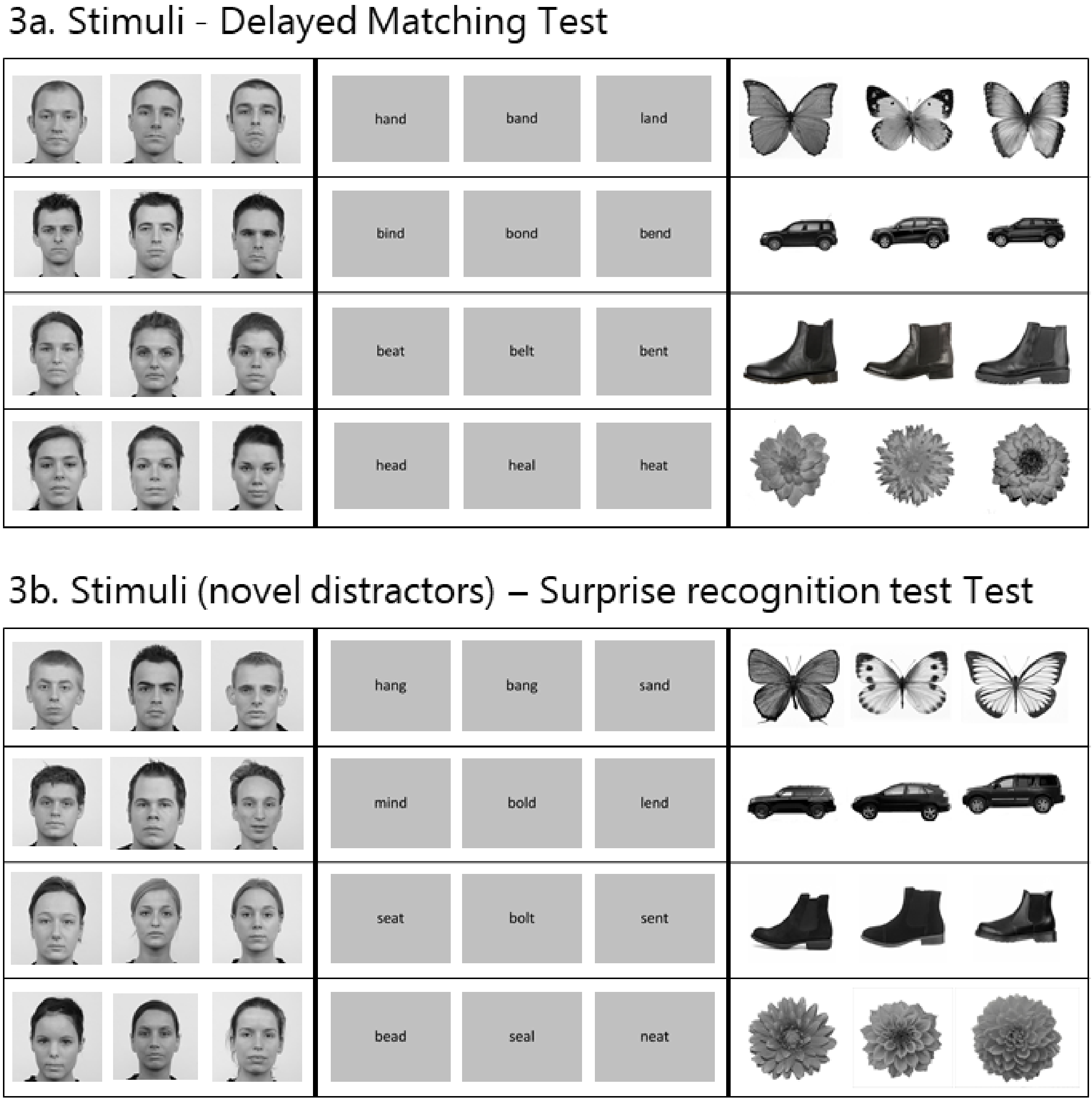

Procedure: The three categories are assessed in separate blocks in the following order: faces, words and objects. A practice session with four practice trials precedes each block. One trial consists of a target stimulus followed by a test stimulus (Supplementary Figure 4a). In 50% of trials, the test stimulus is the same as the target stimulus, and in 50% of trials, the test stimulus is a different stimulus (coming from the same cluster). Participants must determine via button-press whether the test and target stimuli is the same or different.

Accuracy and reaction times are recorded. To avoid the task being a change detection task, test images are presented in smaller dimensions (2/3) than the target images in 50% of trials and in larger dimensions (4/3) than the target images in 50% of trials. Each block (category) involves 48 trials and each cluster of three stimuli is assessed through 12 trials. Trials are presented randomly within a block.

Supplementary Figure 4: Trial outline for the Delayed Matching Test (a), and the Surprise Recognition Test (b).

(a) Trial outline for the Delayed Matching test

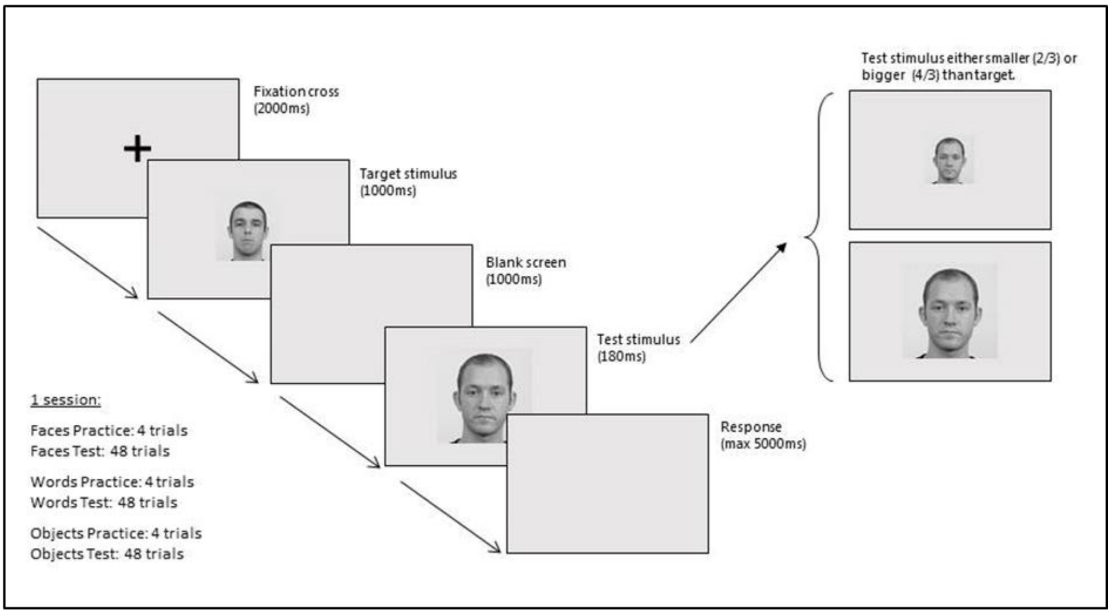

(b) Trial outline for the Surprise Recognition Test

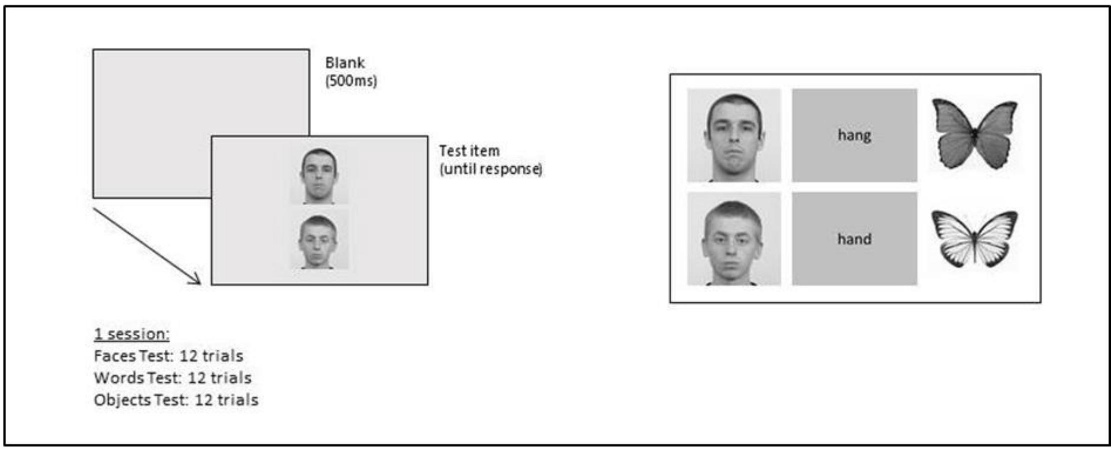

Surprise Recognition Test

Materials: The 36 stimuli used in the *Delayed Matching* part of the test are re-used in this part of the test (Supplementary Figure 3a), 12 novel faces, 12 novel words, and 12 novel objects are also included (Supplementary Figure 3b). The novel stimuli were selected so that they pairwise closely matched the stimuli used in the *Delayed Matching* part of the test. Each new face was selected to look highly similar to a face used in the *Delayed Matching* part of

the test. Each new word differed from the words previously used with one letter only. Each new object was selected to look highly similar to one of the objects previously used. Similarity between images was not formally controlled.

Procedure: The *Surprise Recognition* paradigm is run following a short break after the *Delayed Matching* paradigm. Categories are again presented in separate blocks and are presented in the same order as in the *Delayed Matching* paradigm: faces, words and objects. One trial consisted of a novel face and a target face being presented vertically on a screen. In 50% of trials the target is on top and 50% of trials the target is at the bottom of the screen. Participants are asked to determine which of the images they have seen before by pressing the ↑key or the ↓key. A trial ends when the participant presses a response key (Supplementary Figure 4b). Accuracy and reaction times are recorded. Each target face is presented once. There are therefore 12 trials in each block.

Lexical Decision / Object Decision / Face Familiarity Decision

Reason for inclusion: A task that involves determining whether one has seen a given stimulus before or not was included for each category of interest: words (lexical decision: word or non-word), objects (object decision: object or non-object), and faces (face familiarity: famous or non-famous face). This enables the comparison of visual recognition abilities across categories, without the need for a verbal (naming) output. The lexical decision test involves deciding whether a letter-string stimulus is a word or a non-word, and is commonly used to assess reading abilities (Behrmann & Plaut, 2014; Susilo et al., 2015). The Object Decision test involves determining whether an image is depicting an object or a non-object (Gerlach, 2009). The Famous Face Familiarity Decision test assesses the ability to recognise a face as familiar. Participants must match the perceived face to a representation stored in long term memory. Participants are shown one face at a time and must determine if it is a famous face or a novel face. This task is a measure of perceptual and semantic processing.

Lexical decision - About the test: A 60-item lexical decision task based on Behrmann and Plaut (2014) was administered to assess word recognition. Participants are presented with one stimulus at a time centrally on the screen and must indicate via button-press as quickly and accurately as possible whether the letter-string was a word or not. Items were either 3, 5, or 7 letters in length. Non-words were phonologically plausible letter combinations. The main dependent variables were accuracy and correct response time.

Object decision - About the test: The 72-item test that was included in the BoB protocol has been described in many publications (Gerlach, 2009; Starrfelt et al., 2010). The stimuli are presented one at a time centrally on a screen and participants are required to respond, via button-press, as quickly and accurately as possible whether the stimulus depicts a real object or a (chimeric) nonobject. The main dependent variables were accuracy and correct response time.

Face familiarity decision - About the test: This test contained 80 items, including the 40 famous faces included in the Famous Face Naming task. Faces are presented one at a time centrally on a screen and participants must determine as quickly and accurately as possible via button-press if the face is famous or not. The dependent measures were accuracy and correct response time.

Cambridge Face Memory Test (CFMT)

Reason for inclusion: The CFMT was included as it is the most widely used test for assessing face recognition abilities. This test is highly sensitive to face recognition problems in prosopagnosia (Duchaine & Nakayama, 2006).

About the test: During the test, participants learn a set of 6 new male faces, and then have to recognise them amongst two distractors, either in the presence of visual noise or without (Duchaine & Nakayama, 2006). The dependent measure for this test is accuracy.

Cambridge House Memory Test (CHMT)

Reason for inclusion: The CHMT was designed as a non-face control task for the CFMT (Martinaud et al., 2012). The test assesses house recognition abilities and enables evaluation of the specificity of a participant's face recognition deficits.

About the test: The test has the same experimental set-up as the CFMT but involves learning a set of 6 new houses and then recognising them amongst distractors, either in the presence of visual noise or without (Martinaud et al., 2012). The dependent measure for this test is accuracy.

Word reading (length)

Reason for inclusion: This test measure response time and accuracy when reading words of different lengths and enables calculation of the word length effect, which is a core characteristic of pure alexia. Subjects with hemianopia typically also show a word length effect (although more modest).

About the test: The test has been used in investigations of pure alexia (Habekost et al., 2014; Starrfelt et al., 2009). Participants are asked to read 75 regularly spelled single words out-loud as quickly and accurately as possible. Items are either 3, 5, or 7 letters in length. Each item is displayed on the screen until a response is recorded or a maximum of 4 seconds. Correct response times from stimulus onset to vocal response are measured using a voice key. Accuracy is recorded by the experimenter and responses are recorded using a Dictaphone for the purposes of error analysis.

Word reading: regular, exception, non-word reading

Reason for inclusion: The test measures response time and accuracy when reading non-words, exception words and regular words. The test is included to enable more detailed characterisation of reading deficits in participants with posterior cerebral artery stroke.

About the test: This test includes words (42 regular words, 42 exception words selected from (Patterson & Hodges, 1992) and 20 nonwords (Graham et al., 2000). Participants are presented with words one at a time on the screen and are instructed to read each word out loud as quickly and as accurately as possible. The voice key procedure is the same as that described for the word reading (length) test above. Items are presented on the screen until a response is made or for a maximum of 4 seconds. Accuracy is also recorded by the experimenter.

Naming single letters, digits and 3-letter words

Reason for inclusion: The reasons for inclusion are twofold. To evaluate participants' letter/digit/word naming abilities and to familiarise the participants with the stimuli for the psychophysical single item report experiment.

About the test: This test is a modified version of experiment 2a in (Habekost et al., 2014), which includes single letters and words. A digit condition was added for the BoB protocol to enable analysis of the relationship between letter and digit recognition. Participants are asked to name 30 single letters, 30 single digits, and 30 three-letter words in different blocks. Reaction times and accuracy are measured.

Single item report (digits, letters, and words)

Reason for inclusion: This experiment measures the visual component of letter, word, and digit recognition without being affected by motor components of the response. This is to be compared with the naming task described above, and may indicate if a deficit arises in visual recognition or naming.

About the test: This test is a modified version of experiment 2b in (Habekost et al., 2014), which includes single letters and words, and measures the word superiority effect. A digit condition was added for the BoB protocol to enable analysis of the relationship between letter and digit recognition. The test is a brief version of a psychophysical experiment presenting stimuli (letters, words and digits in separate blocks) at varying, short exposure durations (20, 30, 50, 80 and 100 msec, 10 trials per exposure duration per stimulus type). The dependent measure is overall accuracy across exposure durations, which can be compared between stimulus types. In addition, TVA-based analyses can be carried out on these data, allowing for estimation of perceptual threshold and processing speed for digits, letters, and words respectively (see experiment 2 in (Starrfelt et al., 2013) for analyses of similar data).

Text reading (NEALE)

Reason for inclusion: The *Neale Analysis of Reading Ability* (Neale, 1999*)* measures accuracy, comprehension and rate of reading. It is a standardised test designed to measure reading progression in children and it produces a measure of words read per minute and participants' ability to comprehend what they are reading. The test was used in a recent study involving participants with central alexia (Woodhead et al., 2018). Level one and two texts from the test were included in the BoB protocol to obtain a measure of word and sentence reading comprehension.

About the test: Participants are asked to read two passages of 26 words and 56 words, followed by four and eight comprehension questions, respectively. Participants' responses are recorded using a Dictaphone for transcription of errors and calculation of reading speed.

Picture Naming

Reason for inclusion: A picture naming test that has been used in previous studies (Roberts et al., 2013) was included in the protocol to enable comparison of identification abilities across categories. Picture naming abilities can be compared to famous face naming abilities and word reading abilities.

About the test: Participants were required to name 45 black and white line drawings of objects as quickly and accurately as possible. The stimuli consist of 30 living items (animals, insects) and 15 non-living items (musical instruments, vehicles, tools). Within the living items there is a manipulation of "homomorphy" (Tranel et al., 1997); the amount to which an items contour is shared with other exemplars within that category (15 living items had high homomorphy and 15 living items had low homomorphy). Previous studies have shown that this cohort of patients produce a category effects during naming, in other words performance is worse when naming living items compared to non-living items. The manipulation of homomorphy is included to test the hypothesis that any such category effects are due to low-level perceptual effects caused by the high homomorphy overlap in living items compared to non-living items (which tend to be more unique in their contour). The same voice key procedure described for the word reading tests is adopted. Items are presented on the screen until a response is made or for a maximum of 6 seconds. Accuracy is recorded by the experimenter.

Object Categorisation

Reason for inclusion: An Object Categorisation test was included to measure visual recognition and visual-semantic processing without requiring a verbal (naming) output and to measure category effects. The task requires participants to determine if an object is natural or manmade, and enables the analysis of category effects.

About the test: This test is a short version of the object categorisation tasks used by e.g. (Gerlach et al., 2016). Stimuli are 36 Snograss and Vanderwart line drawings: 18 representing natural objects and 18 representing man-made objects. Images are presented one at a time and participants are required to respond via button-press as quickly and accurately as possible whether it depicts a natural or a manmade object. The dependent variables are correct response time and accuracy.

Famous Face Naming

Reason for inclusion: A Famous Face Naming test was included to enable comparison of identification abilities across categories. Famous Face Naming abilities can be compared to Picture Naming Abilities and Word Reading abilities.

About the test: This test was used in a previous case-series investigation of posterior cerebral lesions (Roberts et al., 2015). The test contains 40 items, pictures of famous faces are presented one at a time centrally on a screen and participants are asked to name the person out loud as quickly and as accurately as possible. If they are unable to provide their name, recognition of the person is tested (e.g., provision of why the person is famous, what they do, where they live etc.). The main measure for this test is accuracy, reaction time data is not scored due to the extensive verbal output. Responses are scored according to whether the correct name is provided and whether correct semantic information is provided. The items included in this test were also included in the Face Familiarity test (the two tests were administered on different days, face familiarity first).

Associated functions

Writing to dictation

Reason for inclusion: A writing to dictation test was included in the protocol to establish whether the participants with alexia have intact spelling.

About the test: The original writing to dictation test includes 80 items (Graham, Patterson, & Hodges, 2000) and to make the test shorter and harder, only the 40 low predictability words were included in the BoB protocol (alongside 20 non-words (Graham et al., 2000)). The experimenter reads each item aloud to the participant, who then has to repeat each word back, and then write the item down on a score sheet. The experimenter measures two reaction times: (1) planning time: measured as the time from correct repetition to the time writing began, (2) writing time: measured from the start of writing to the end. The main dependent variables are accuracy and the two measures of response time.

Surprise Handwriting Test

Reason for inclusion: A Surprise Handwriting Recognition Test was included to assess whether participants can read something that they have written and whether they can recognise their own handwriting. Early case studies of Pure Alexia describe participants as unable to read something they have written a short time previously (Bub et al., 1993). Also, while reading is generally considered to be supported by processes that are left lateralised, some studies suggest that the recognition of handwriting style may be right lateralised and may occur together with face processing deficits (Hills et al., 2015). Assessing handwriting recognition enables investigation of the lateralisation of such deficits.

About the test: This test was devised for the purposes of the current study. On the first day of testing participants are asked to write a simple sentence taken from a level one passage in the Neale (Neale, 1999). Handwritten samples of the remaining sentences within this Neale passage were obtained prior to testing. The participants' writing is scanned into the computer and inserted as the second sentence within the Neale passage. The participants are presented with a level one passage from the Neale to read in four different handwritings, one of which is their own. Three measures are obtained; i) the time taken to read the passage, ii) whether the participant spontaneously recognises the handwriting as their own, and iii) whether the participant is able to identify the handwriting as their own upon forced choice.

Synonym Judgement Task

Reason for inclusion: The Synonym Judgement Task is one of the standard tests assessing semantic memory. The test can be used to explore even mild semantic problems (impaired accuracy on the hardest, lower frequency items). The test was included to determine whether a participant's recognition problem can be explained, at least partially, by semantic problems.

About the test: The task has been used in many previous studies, including with other patient groups (Jefferies et al., 2009), and healthy participants (Binney et al., 2010). The task includes 96 items. For each trial a written target word is presented on screen, alongside three choices. Participants are instructed to determine which of the choice items is associated with the target item. Stimulus presentation was adjusted for the BoB project so words are presented vertically rather than horizontally. Stimuli are presented visually and as spoken words, to avoid biasing against patients who struggled to read. The experimenter reads each word out loud and points to each word on the screen before prompting a response (for this reason reaction times are not collected on this task). Stimuli vary according to imageability (high vs. low) as well as frequency (high, medium, low).

Full list of tests included in the Back of the Brain project and how to access them

Tests are presented in the order in which they were administered in the project.

Order	Test	Original reference	How to access test
1	Edinburgh short-form 5 item	(Veale, 2014)	In original publication https://doi.org/10.1080/1357650x.2013.783045
2	Geriatric Depression Scale - 15	(Yesavage & Sheikh, 1986)	In original publication https://doi.org/10.1300/J018v05n01_09
3	Oxford Cognitive Screen	(Demeyere et al., 2015)	www.innovation.ox.ac.uk
4	Digit span forwards, backwards WAIS IV.	(Wechsler, 2010)	www.pearson.com/uk/
5	Basic motor response	Designed for project	Available from Ro J. Robotham: jer@psy.ku.dk
6	Face recognition questionnaire	(Freeman et al., 2015)	Original test (open access): https://doi.org/10.6084/m9.figshare.1501441.v1 Items inlcuded and amended for the current project listed in text.
7	Wayfinding questionnaire	(Claessen et al., 2016)	Original test in original publication (open access) https://doi.org/10.1093/arclin/acw044 Items selected for current project listed in text.
8	Delayed Matching and Surprise Recognition test (WOF test)	(Robotham, 2019)	Available from Ro J. Robotham: jer@psy.ku.dk
9	Adult reading history questionnaire	(Lefly & Pennington, 2000)	In original publication: https://doi.org/10.1177/002221940003300306
10	Famous Face Familiarity decision	Designed for project	Available from Matt.Lambon-Ralph@mrc-cbu.cam.ac.uk
11	Synonym judgement task	(Jefferies et al., 2009)	Available from Matt.Lambon-Ralph@mrc-cbu.cam.ac.uk
12	Leuven Perceptual Organisation Screening test (modified)	(Torfs et al., 2014)	Original version: http://www.gestaltrevision.be/tests/index.php?language=en Modified version available from Randi Starrfelt (Randi.Starrfelt@psy.ku.dk) or jer@psy.ku.dk (Ro J. Robotham)
13	Writing to dictation	(Graham et al., 2000)	List of selected stimuli available from Randi.Starrfelt@psy.ku.dk

14	Cambridge Face Memory Test	(Duchaine & Nakayama, 2006)	Contact authors of original publication https://doi.org/10.1016/j.neuropsychologia.2005.07.001
15	Object decision	(Gerlach, 2009)	Available from Randi.Starrfelt@psy.ku.dk
16	Cambridge house memory test	(Martinaud et al., 2012)	Contact authors of original publication https://doi.org/10.1371/journal.pone.0030433
17	Lexical decision	(Behrmann & Plaut, 2014)	Available from Randi.Starrfelt@psy.ku.dk
18	Computerised Visual Field Test	(Nordfang et al., 2019)	Freely available: https://psy.ku.dk/ansatte/?pure=da%2Fpublications%2Fcvft(c39487b2-2ad1-4cb0-bf6d-73d79eb87a06).html
19	Naming single letters, digits and 3-letter words	(Habekost et al., 2014)	Available from Randi.Starrfelt@psy.ku.dk
20	Single item report: letters, digits and words	(Habekost et al., 2014)	Available from Randi.Starrfelt@psy.ku.dk
21	FrACT - Visual acuity	(Bach, 1996)	www.michaelbach.de/fract
22	FACT - Contrast sensitivity	(Ginsburg, 1984)	www.stereooptical.com/
23	Word reading (length: 3,5,7 letter words)	(Starrfelt et al., 2009)	Available from Randi.Starrfelt@psy.ku.dk
24	Picture naming	(Roberts et al., 2013)	Available from Matt.Lambon-Ralph@mrc-cbu.cam.ac.uk
25	Famous face naming	(Roberts et al., 2015)	Available from Matt.Lambon-Ralph@mrc-cbu.cam.ac.uk
26	Word reading: regular, irregular and non-words	(Graham et al., 2000; Patterson & Hodges, 1992)	Available from Randi.Starrfelt@psy.ku.dk
27	Farnsworth D-15 test of colour perception	(Linksz, 1966)	Test copyrighted. Various retailers.
28	NEALE: text reading	(Neale, 1999)	Test copyrighted. Various retailers.
29	Surprise Handwriting recognition test	Created for current project	Available from Randi.Starrfelt@psy.ku.dk

30	Object categorisation	(Gerlach, 2009)	Available from Randi.Starrfelt@psy.ku.dk

Supplementary Note 2: Stability of the PCA solution

To assess the stability of the PCA solution shown in Figure 2, two different approaches were taken: (1) use of an alternative clustering method (hierarchal cluster analysis) on the tests of lower-and higher-level visual processing, and (2) PCA including only the higher-level tests of visual processing.

Hierarchal cluster analysis of the tests of lower and higher-level visual processing

(a) Varimax-rotated PCA solution of lower- and higher-level visual tests

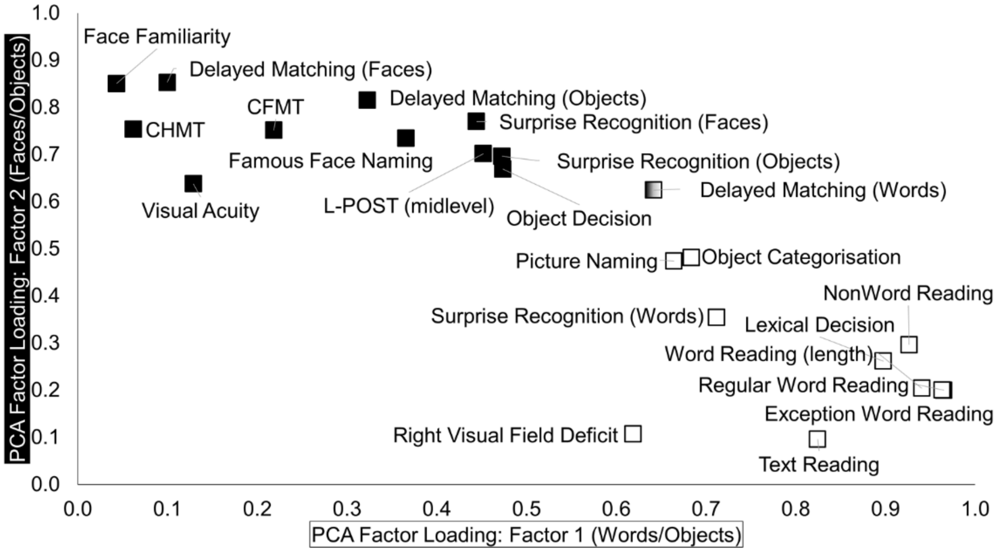

(b) Hierarchal cluster analysis of lower- and higher-level visual tests

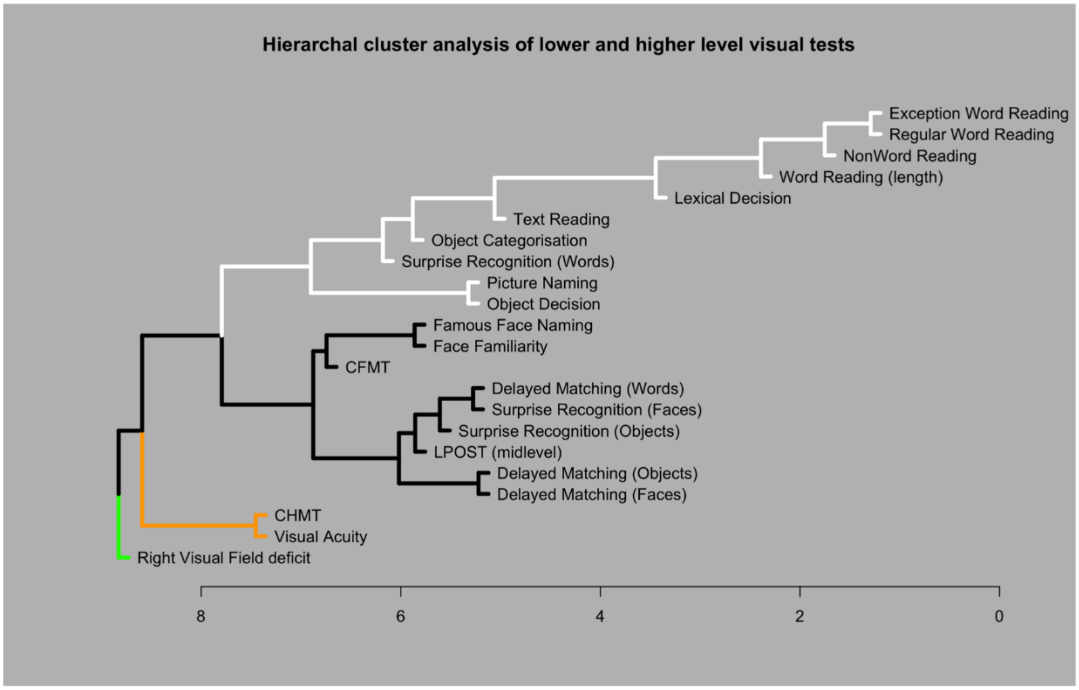

Supplementary Figure 5: Comparison between the varimax-rotated solution of the lower- and higher-order visual tests and a hierarchal cluster analysis of the same data. Tests which load significantly on PCA Factor 1 (Word/Object) are highlighted in white, tests which load on Factor 2 (Faces/Object) are highlighted in black in both panels.

To assess whether Factor 2 ("Faces/Object") would further subdivide into tests of face processing and object processing, hierarchal cluster analysis was used. Taking the same input data to the PCA model, Supplementary Figure 5 shows that there was no further fine-grained splitting of object category in the Faces/Object factor. Hierarchal cluster analysis replicated the main division between the tests of word recognition/reading and the face/object processing tests. Within the face/object cluster, tests were split according to test difficulty rather than object category.

PCA of higher-order visual tests

To assess the stability of the effects shown in Figure 2 we conducted the PCA including only the tests of word, object, and face recognition listed in Table 2. The varimax rotated PCA of the tests of higher-level visual perception again produced two principal factors exceeding an eigenvalue of 1. These two factors explained 77% of the variance of the original data with a KMO of 0.893.

Supplementary Figure 6a illustrates how each neuropsychological test loads on these two principal factors – the organisation of both factors remained identical to the solution including tests of lower-level visual processing. The only difference was that the order of the principal factors changed, such that Factor 1 in the original analysis in Figure 2 represents the tests of face and object processing, whereas here it represents the tests of word recognition. Factor 1 accounted for 63% of the variance and contained all tests of word processing, in particular reading. Factor 2 accounted for 14% of the variance and contained all tests of face processing and some tests of object processing (Object Decision, CHMT).

Supplementary Figure 6b displays how each of the 64 patients performed on the two principal factors. The distribution of patients along each principal factor also did not vary from the solution presented in Figure 2.

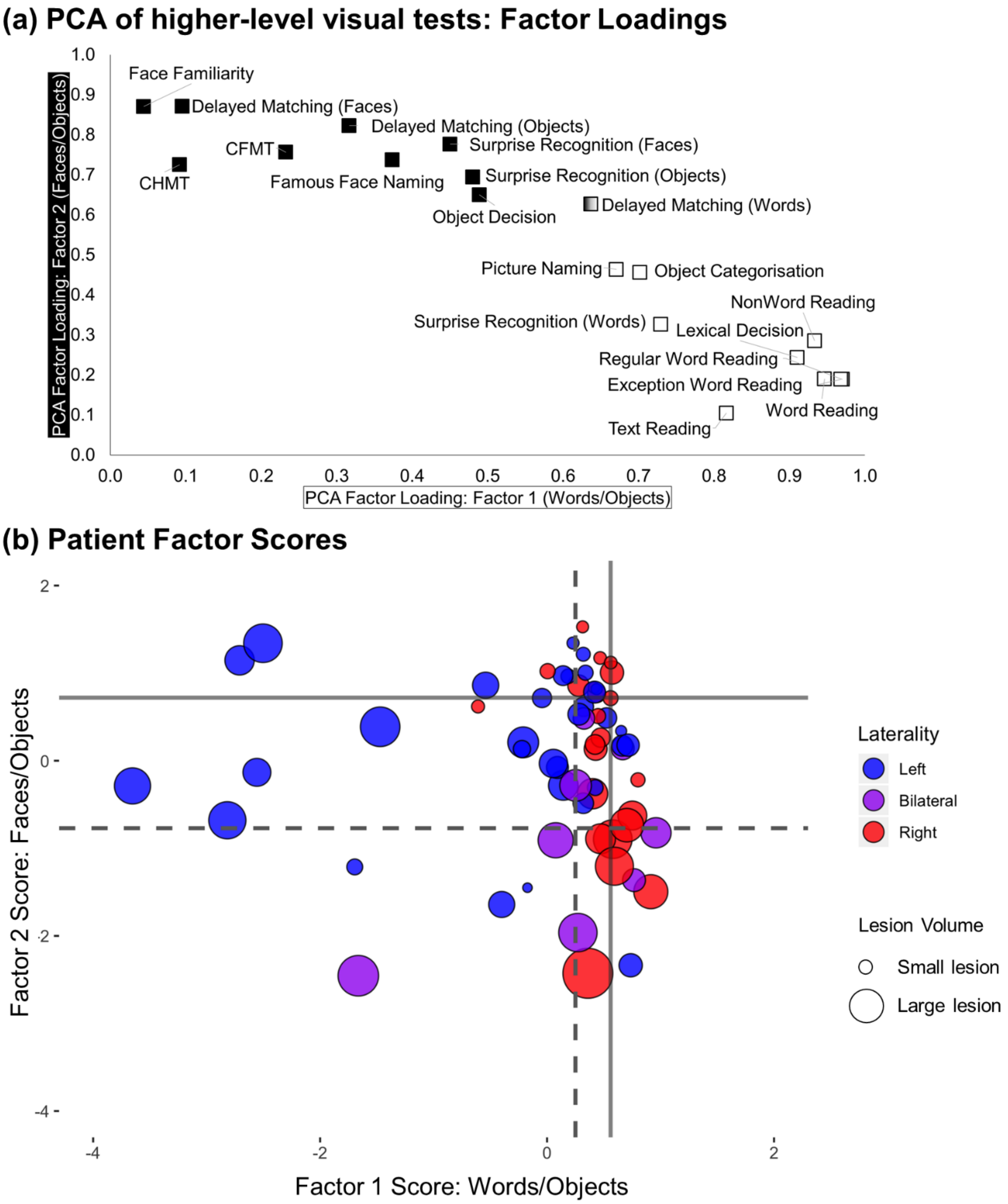

Supplementary Figure 6: Varimax rotated PCA of higher-level visual tests. (a) A two-factor solution explained 77% of the variance, with a KMO of 0.893. Each point represents the factor loading of each neuropsychological test on the two principle factors extracted from the data. Tests that load significantly on Factor 1 (Words/Objects) are shown in white, tests which load

significantly on Factor 2 (Faces/Objects) shown in black. (b) Patient factor scores on the two principal factors extracted in the data-driven analysis. Each point represents one patient. Points are colour coded according to lesion laterality (left hemisphere strokes in blue, right hemisphere strokes in red, bilateral strokes in purple). The size of each point denotes the size of the stroke (larger points = larger stroke volume). The solid lines represent the average control group performance on each factor. The dashed lines represent two standard deviations away from the control mean for each factor.

Supplementary Note 3: Composite Score Analysis

Composite Score Factor Loading Tables

Supplementary Table 2: Factor Loading tables for the unrotated, fixed factor PCA used to generate the Composite Scores. Tests/measures which significantly loaded with the factor are marked in bold.

Domain	Test	Measure	Factor Loading
**Words**	Word Reading	Accuracy	**0.916**
	Word Reading	Reaction Time	**0.908**
	Lexical Decision	Reaction Time	**0.873**
	Delayed Matching (Words)	Accuracy	**0.870**
	Surprise Recognition (Words)	Reaction Time	**0.865**
	Lexical Decision	Accuracy	**0.858**
	Delayed Matching (Words)	Reaction Time	**0.814**
	Surprise Recognition (Words)	Accuracy	**0.514**
**Objects**	Object Decision	Accuracy	**0.854**
	Delayed Matching (Objects)	Reaction Time	**0.799**
	Surprise Recognition (Objects)	Reaction Time	**0.793**
	Picture Naming	Accuracy	**0.775**
	Delayed Matching (Objects)	Accuracy	**0.754**
	Object Decision	Reaction Time	**0.738**
	Surprise Recognition (Objects)	Accuracy	**0.688**
	Picture Naming	Reaction Time	0.401
**Faces**	Famous Face Recognition	Accuracy	**0.894**
	Famous Face Naming	Accuracy	**0.849**
	Delayed Matching (Faces)	Accuracy	**0.841**
	Surprise Recognition (Faces)	Reaction Time	**0.822**
	Face Familiarity	Accuracy	**0.810**
	Face Familiarity	Reaction Time	**0.808**
	Surprise Recognition (Faces)	Reaction Time	**0.806**
	Delayed Matching (Faces)	Reaction Time	**0.718**

Dissociation Analysis

Supplementary Table 3: Pattern of significant deficits and dissociations in the composite score analysis, including age as a covariate. Deficit analyses were run on all patients using the BDT_Cov_Raw.exe toolbox (Crawford et al., 2011). Dissociation analyses were run on any patient who showed at least one significant deficit in any domain, using the BDST_Cov_Raw.exe toolbox (Crawford et al., 2011). Deficits patterns are coloured coded

according to Figure 3b. The domain which is listed first indicates the strongest deficit (e.g., W > F indicates that performance on words is significantly worse than performance on faces).

Deficit Pattern	Dissociation Pattern	Left	Bilateral	Right	Total
**WOF**		**10**	**6**	**6**	**22**
	None	2	2	2	6
	W > F	1	1	0	2
	W > O	1	0	0	1
	O > F	0	0	1	1
	W > F / W > O	4	1	2	7
	W > O / O > F	0	0	1	1
	W > F / W > O / O > F	2	2	0	4
**W only**		**3**	**0**	**3**	**6**
	W > O	0	0	1	1
	W > F / W > O	3	0	2	5
**WF**		**2**	**0**	**1**	**3**
	None	0	0	1	1
	W > O	1	0	0	1
	W > O / F > W	1	0	0	1
**WO**		**5**	**0**	**4**	**9**
	None	0	0	1	1
	W > F	0	0	1	1
	W > F / W > O	4	0	0	4
	O > F / W > O	0	0	1	1
	W > F / O > F	0	0	1	1
	W > F / W > O / O > F	1	0	0	1
**OF**		**0**	**2**	**1**	**3**
	None	0	1	0	1
	O > W	0	0	1	1
	F > W / O > F	0	1	0	1

Non-parametric equivalent of the Composite Score Analysis

As an alternative to generating the composite scores using unrotated PCA, composite scores were also generated using ranks. For this method, each subject's (controls and patients) raw score performance on each test measure was given a rank using the 'rank.avg' function in Excel (using the ascending method, such that lower ranks indicate poorer performance). For each measure, the ranks were normalised based on the number of patients completing that test (i.e.,

to account for missing data). This generated a rank between 0 and 1 on each test measure. Then composite ranks for each domain were generated by averaging each subjects ranks across the comparable tests listed in Table 2.

The same statistical comparisons as described in the main methods were carried out to compare each patients performance in each of the three domains to controls performance (accounting for age).

Supplementary Figure 7a summarises the raw composite scores across the two methods. The main difference between the two methods were that the rank analysis produced much greater variability (even in the control group). This was because the ranks were a lot more unique, and did not take into account the variability between individual patients. For example, patient 1 and patient 2 could perform very differently from each other, but still get consecutive ranks. In the unrotated PCA, these relative differences were preserved.

(a) Raw Composite Scores

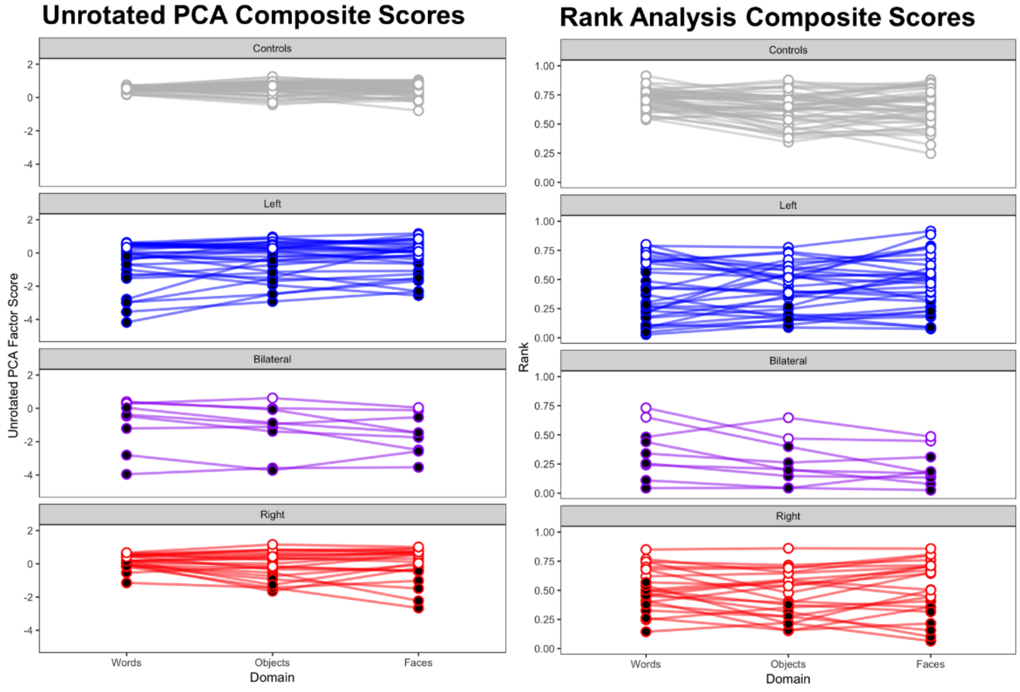

(b) Patterns of deficits across the patient group

Unrotated PCA Composite Scores

Deficit Pattern	Left	Bilateral	Right	Total
**No Deficits**	12	1	8	21
**WOF**	10	6	6	22
**W only**	3	0	3	6
O only	*0*	*0*	*0*	*0*
F only	*0*	*0*	*0*	*0*
**WF**	2	0	1	3
**OF**	0	2	1	3
**WO**	5	0	4	9

Rank Analysis Composite Scores

Deficit Pattern	Left	Bilateral	Right	Total
**No Deficits**	10	1	7	18
**WOF**	10	6	7	23
**W only**	7	1	4	12
O only	*0*	*0*	*0*	*0*
**F only**	1	0	1	2
**WF**	1	0	1	2
**OF**	0	1	0	1
**WO**	3	0	3	6

Supplementary Figure 7: Comparison of the composite score analysis conducted using unrotated PCA (left: as presented in Figure 3), compared to a non-parametric equivalent using ranks (right). (a) In both methods, lower scores indicate worse performance. Patients showing a statistically significant deficit in a domain, compared to the control group are highlighted in black circles. (b) Showing the pattern of deficits based on the number of statistically significant modified t-tests per patient.

Supplementary Figure 7b compares the patterns of deficits seen in the patient group across both composite score methods. The pattern of deficits seen in the patient group are broadly similar across the two composite score methods (11 patients deficit pattern changed between analyses). As in the main analysis, the predominant pattern in the non-parametric, ranking

method was either deficits across all domains, or in none. Selective deficits are still rare, but most commonly found is a category-selective deficit for word recognition.

The additional patients who showed a category selective deficit for words in the rank analysis, also show a deficit for objects in the PCA analysis. There are additional patients who show a word-selective deficit in the rank analysis, but in the PCA they change to having no deficits (3 patients), having a dual-deficit for words and objects (2 patients), or having a dual-deficit for words and faces (1 patient).

The biggest differences between the two analysis methods is that in the rank analysis there are patients who show a category-selective deficit for faces (orange). In the unrotated PCA these results change to show no deficits in one patient, and a dual deficit for faces and objects in the second.

References

Bach, M. (1996). The Freiburg Visual Acuity test - Automatic measurement of visual acuity. *Optometry and Vision Science*, *73*(1), 49-53. https://doi.org/10.1097/00006324-199601000-00008

Behrmann, M., & Plaut, D. C. (2014). Bilateral Hemispheric Processing of Words and Faces: Evidence from Word Impairments in Prosopagnosia and Face Impairments in Pure Alexia [Article]. *Cerebral Cortex*, *24*(4), 1102-1118. https://doi.org/10.1093/cercor/bhs390

Binney, R. J., Embleton, K. V., Jefferies, E., Parker, G. J., & Lambon Ralph, M. A. (2010). The ventral and inferolateral aspects of the anterior temporal lobe are crucial in semantic memory: evidence from a novel direct comparison of distortion-corrected fMRI, rTMS, and semantic dementia. *Cerebral Cortex*, *20*(11), 2728-2738. http://www.ncbi.nlm.nih.gov/pubmed/20190005

Bouvier, S. E., & Engel, S. A. (2006). Behavioral deficits and cortical damage loci in cerebral achromatopsia. *Cerebral Cortex*, *16*(2), 183-191. https://doi.org/10.1093/cercor/bhi096

Bub, D. N., Arguin, M., & Lecours, A. R. (1993). Dejerine, Jules and his interpretation of pure alexia. *Brain and Language*, *45*(4), 531-559. https://doi.org/10.1006/brln.1993.1059

Claessen, M. H. G., Visser-Meily, J. M. A., de Rooij, N. K., Postma, A., & van der Ham, I. J. M. (2016). The Wayfinding Questionnaire as a Self-report Screening Instrument for Navigation-related Complaints After Stroke: Internal Validity in Healthy Respondents and Chronic Mild Stroke Patients. *Archives of Clinical Neuropsychology*, *31*(8), 839-854. https://doi.org/10.1093/arclin/acw044

Crawford, J. R., Garthwaite, P. H., & Ryan, K. (2011). Comparing a single case to a control sample: Testing for neuropsychological deficits and dissociations in the presence of covariates. *Cortex*, *47*(10), 1166-1178. https://doi.org/10.1016/j.cortex.2011.02.017

Demeyere, N., Riddoch, M. J., Slavkova, E. D., Bickerton, W. L., & Humphreys, G. W. (2015). The Oxford Cognitive Screen (OCS): Validation of a Stroke-Specific Short Cognitive Screening Tool. *Psychological Assessment*, *27*(3), 883-894. https://doi.org/10.1037/pas0000082

Duchaine, B., & Nakayama, K. (2006). The Cambridge Face Memory Test: results for neurologically intact individuals and an investigation of its validity using inverted face stimuli and prosopagnosic participants. *Neuropsychologia*, *44*(4), 576-585. https://doi.org/10.1016/j.neuropsychologia.2005.07.001

Farnsworth, D. (1943). The Farnsworth-Munsell 100-hue and dichotomous tests for color vision. *Journal of the Optical Society of America*, *33*(10), 568-578. https://doi.org/10.1364/josa.33.000568

Freeman, P., Palermo, R., & Brock, J. (2015). *Faces and Emotion Questionnaire*. https://doi.org/10.6084/m9.figshare.1501441.v1
Gerlach, C. (2009). Category-specificity in visual object recognition. *Cognition*, *111*(3), 281-301. https://doi.org/10.1016/j.cognition.2009.02.005
Gerlach, C., Klargaard, S. K., & Starrfelt, R. (2016). On the Relation between Face and Object Recognition in Developmental Prosopagnosia: No Dissociation but a Systematic Association. *Plos One*, *11*(10), Article e0165561. https://doi.org/10.1371/journal.pone.0165561
Ginsburg, A. P. (1984). A new contrast sensitivity vision test chart. *Am J Optom Physiol Opt*, *61*(6), 403-407. https://doi.org/10.1097/00006324-198406000-00011
Graham, N. L., Patterson, K., & Hodges, J. R. (2000). The impact of semantic memory impairment on spelling: evidence from semantic dementia. *Neuropsychologia*, *38*(2), 143-163. https://doi.org/https://doi.org/10.1016/S0028-3932(99)00060-3
Habekost, T., Petersen, A., Behrmann, M., & Starrfelt, R. (2014). From word superiority to word inferiority: visual processing of letters and words in pure alexia. *Cognitive Neuropsychology*, *31*(5-6), 413-436. https://doi.org/10.1080/02643294.2014.906398
Hackett, M. L., Yapa, C., Parag, V., & Anderson, C. S. (2005). Frequency of depression after stroke - A systematic review of observational studies. *Stroke*, *36*(6), 1330-1340. https://doi.org/10.1161/01.str.0000165928.19135.35
Hills, C. S., Pancaroglu, R., Duchaine, B., & Barton, J. J. S. (2015). Word and text processing in acquired prosopagnosia. *Annals of Neurology*, *78*(2), 258-271. https://doi.org/10.1002/ana.24437
Jefferies, E., Patterson, K., Jones, R. W., & Lambon Ralph, M. A. (2009). Comprehension of Concrete and Abstract Words in Semantic Dementia [Article]. *Neuropsychology*, *23*(4), 492-499. https://doi.org/10.1037/a0015452
Langner, O., Dotsch, R., Bijlstra, G., Wigboldus, D. H. J., Hawk, S. T., & van Knippenberg, A. (2010). Presentation and validation of the Radboud Faces Database. *Cognition & Emotion*, *24*(8), 1377-1388. <Go to ISI>://WOS:000284609100006
Lefly, D. L., & Pennington, B. F. (2000). Reliability and validity of the adult reading history questionnaire. *Journal of Learning Disabilities*, *33*(3), 286-296. https://doi.org/10.1177/002221940003300306
Linksz, A. (1966). Farnsworth panel D-15 test. *American Journal of Ophthalmology*, *62*(1), 27-&. https://doi.org/10.1016/0002-9394(66)91673-4
Martinaud, O., Pouliquen, D., Gerardin, E., Loubeyre, M., Hirsbein, D., Hannequin, D., & Cohen, L. (2012). Visual Agnosia and Posterior Cerebral Artery Infarcts: An Anatomical-Clinical Study. *Plos One*, *7*(1), Article e30433. https://doi.org/10.1371/journal.pone.0030433
Neale, M. D. (1999). *Neale Analysis of Reading Ability* (3rd ed ed.). Melbourne: ACER.
Nordfang, M., Uhre, V., Robotham, R. J., Kerry, S. J., Frederiksen, J. L., & Starrfelt, R. (2019). A free and simple computerized screening test for visual field defects. *Scandinavian Journal of Psychology*, *60*(4), 289-294. https://doi.org/10.1111/sjop.12546
Oldfield, R. C. (1971). The assessment and analysis of handedness: The Edinburgh Inventory. *Neuropsychologia*, *9*(1), 97-113. https://doi.org/10.1016/0028-3932(71)90067-4
Patterson, K., & Hodges, J. R. (1992). Deterioration of word meaning: Implications for reading. *Neuropsychologia*, *30*(12), 1025-1040. https://doi.org/https://doi.org/10.1016/0028-3932(92)90096-5
Roberts, D. J., Lambon Ralph, M. A., Kim, E., Tainturier, M. J., Beeson, P. M., Rapcsak, S. Z., & Woollams, A. M. (2015). Processing deficits for familiar and novel faces in patients with left posterior fusiform lesions. *Cortex*, *72*, 79-96. https://doi.org/10.1016/j.cortex.2015.02.003
Roberts, D. J., Woollams, A. M., Kim, E., Beeson, P. M., Rapcsak, S. Z., & Lambon Ralph, M. A. (2013). Efficient Visual Object and Word Recognition Relies on High Spatial Frequency Coding in the Left Posterior Fusiform Gyrus: Evidence from a Case-Series of Patients with Ventral Occipito-

Temporal Cortex Damage. *Cerebral Cortex*, *23*(11), 2568-2580. https://doi.org/10.1093/cercor/bhs224

Robotham, R. J. (2019). *The Neuropsychology of Stroke in the Back of the Brain: Clinical and Cognitive Aspects.* University of Copenhagen].

Starrfelt, R., Gerlach, C., Habekost, T., & Leff, A. P. (2013). Word-superiority in pure alexia. *Behavioural Neurology*, *26*(3), 167-169. https://doi.org/10.3233/ben-2012-129002

Starrfelt, R., Habekost, T., & Gerlach, C. (2010). Visual processing in pure alexia: A case study. *Cortex*, *46*(2), 242-255. https://doi.org/10.1016/j.cortex.2009.03.013

Starrfelt, R., Habekost, T., & Leff, A. P. (2009). Too Little, Too Late: Reduced Visual Span and Speed Characterize Pure Alexia. *Cerebral Cortex*, *19*(12), 2880-2890. https://doi.org/10.1093/cercor/bhp059

Susilo, T., Wright, V., Tree, J. J., & Duchaine, B. (2015). Acquired prosopagnosia without word recognition deficits. *Cognitive Neuropsychology*, *32*(6), 321-339. https://doi.org/10.1080/02643294.2015.1081882

Torfs, K., Vancleef, K., Lafosse, C., Wagemans, J., & de-Wit, L. (2014). The Leuven Perceptual Organization Screening Test (L-POST), an online test to assess mid-level visual perception. *Behavior Research Methods*, *46*(2), 472-487. https://doi.org/10.3758/s13428-013-0382-6

Tranel, D., Damasio, H., & Damasio, A. R. (1997). A neural basis for the retrieval of conceptual knowledge. *Neuropsychologia*, *35*(10), 1319-1327. http://www.ncbi.nlm.nih.gov/pubmed/9347478

Vancleef, K., Acke, E., Torfs, K., Demeyere, N., Lafosse, C., Humphreys, G., Wagemans, J., & de-Wit, L. (2015). Reliability and validity of the Leuven Perceptual Organization Screening Test (L-POST). *Journal of Neuropsychology*, *9*(2), 271-298. https://doi.org/10.1111/jnp.12050

Veale, J. F. (2014). Edinburgh Handedness Inventory - Short Form: A revised version based on confirmatory factor analysis. *Laterality*, *19*(2), 164-177. https://doi.org/10.1080/1357650x.2013.783045

Wechsler, D. (2010). *Wechsler Adult Intelligence Scale - IV UK*. Pearson Clinical UK.

Woodhead, Z. V. J., Kerry, S. J., Aguilar, O. M., Ong, Y. H., Hogan, J. S., Pappa, K., Leff, A. P., & Crinion, J. T. (2018). Randomized trial of iReadMore word reading training and brain stimulation in central alexia. *Brain*, *141*, 2127-2141. https://doi.org/10.1093/brain/awy138

Yesavage, J. A., Brink, T. L., Rose, T. L., Lum, O., Huang, V., Adey, M., & Leirer, V. O. (1983). Development and validation of a geriatric depression screening scale - a preliminary report. *Journal of Psychiatric Research*, *17*(1), 37-49. https://doi.org/10.1016/0022-3956(82)90033-4

Yesavage, J. A., & Sheikh, J. I. (1986). Geriatric Depression Scale (GDS) Recent Evidence and Development of a Shorter Version. *Clinical Gerontologist*, *5*(1-2), 165-173. https://doi.org/10.1300/J018v05n01_09
